# Activity of translation regulator eukaryotic elongation factor-2 kinase is increased in Parkinson disease brain and its inhibition reduces alpha synuclein toxicity

**DOI:** 10.1186/s40478-018-0554-9

**Published:** 2018-07-02

**Authors:** Asad Jan, Brandon Jansonius, Alberto Delaidelli, Forum Bhanshali, Yi Andy An, Nelson Ferreira, Lisa M. Smits, Gian Luca Negri, Jens C. Schwamborn, Poul H. Jensen, Ian R. Mackenzie, Stefan Taubert, Poul H. Sorensen

**Affiliations:** 1 Aarhus Institute of Advanced Studies, Department of Biomedicine, Aarhus University, Høegh-Guldbergs Gade 6B, DK-8000 Aarhus, Denmark; 2 Department of Pathology and Laboratory Medicine, University of British Columbia, Vancouver, Canada; 3 British Columbia Cancer Research Centre, 675 West 10th Avenue, Vancouver, BC V5Z 1L3 Canada; 4 Centre for Molecular Medicine and Therapeutics, BC Children’s Hospital Research Institute, Department of Medical Genetics, University of British Columbia, Vancouver, BC V5Z 4H4 Canada; 5 Danish Research Institute of Translational Neuroscience, Department of Biomedicine, Aarhus University, Ole Worms Allé 3, DK-8000 Aarhus, Denmark; 6 Developmental and Cellular Biology, Luxembourg Centre for Systems Biomedicine (LCSB), University of Luxembourg, 7, avenue des Hauts-Fourneaux, 4362 Esch-sur-Alzette, Luxembourg

**Keywords:** eEF2K, Parkinson disease, Alpha synuclein, Oxidative stress, Neurotoxicity

## Abstract

Parkinson disease (PD) is the second most common neurodegenerative disorder and the leading neurodegenerative cause of motor disability. Pathologic accumulation of aggregated alpha synuclein (AS) protein in brain, and imbalance in the nigrostriatal system due to the loss of dopaminergic neurons in the substantia nigra- pars compacta, are hallmark features in PD. AS aggregation and propagation are considered to trigger neurotoxic mechanisms in PD, including mitochondrial deficits and oxidative stress. The eukaryotic elongation factor-2 kinase (eEF2K) mediates critical regulation of dendritic mRNA translation and is a crucial molecule in diverse forms of synaptic plasticity. Here we show that eEF2K activity, assessed by immuonohistochemical detection of eEF2 phosphorylation on serine residue 56, is increased in postmortem PD midbrain and hippocampus. Induction of aggressive, AS-related motor phenotypes in a transgenic PD M83 mouse model also increased brain eEF2K expression and activity. In cultures of dopaminergic N2A cells, overexpression of wild-type human AS or the A53T mutant increased eEF2K activity. eEF2K inhibition prevented the cytotoxicity associated with AS overexpression in N2A cells by improving mitochondrial function and reduced oxidative stress. Furthermore, genetic deletion of the eEF2K ortholog *efk-1* in *C. elegans* attenuated human A53T AS induced defects in behavioural assays reliant on dopaminergic neuron function. These data suggest a role for eEF2K activity in AS toxicity, and support eEF2K inhibition as a potential target in reducing AS-induced oxidative stress in PD.

**Electronic supplementary material:**

The online version of this article (10.1186/s40478-018-0554-9) contains supplementary material, which is available to authorized users.

## Introduction

Parkinson disease (PD) is the most common neurodegenerative cause of motor disability and is estimated to affect around 10 million people worldwide [33, 53]. Clinically, it presents as a movement disorder characterized by resting tremor, rigidity, and bradykinesia, and in a substantial number of patients the motor disability is compounded by non-motor symptoms such as cognitive impairment and autonomic dysfunction [33, 53]. Neuropathologically, loss of dopamine producing neurons in the midbrain *substantia nigra* (SN)-*pars compacta*, and intraneuronal inclusions of aggregated α-synuclein (AS) protein in multiple brain regions are hallmark features in PD [33, 53]. AS is a 14 kDa cytosolic protein (encoded by the *SNCA* gene) with putative roles in synaptic vesicle recycling, mitochondrial functions, and chaperone activity [39, 71]. Deposition of AS in the form of inclusions in neurons and/or nerve terminals, also known as Lewy body pathology, is also seen in other neurodegenerative diseases such as Alzheimer disease (AD), Lewy body dementia (LBD), and in oligodendrocytes in Multiple system atrophy (MSA) [67]. Idiopathic (non-inheritable) PD accounts for a vast majority of cases, while 5–10% of clinically diagnosed PD is attributable to genetic factors [53]. Missense mutations in *SNCA* resulting in N-terminal amino acid substitutions in the AS protein, or multiplications in *SNCA* gene locus leading to increased AS expression are the earliest known causes of autosomal-dominant inherited forms of PD [53, 54, 62]. There are additional genes associated with familial PD including autosomal-dominant and recessive inheritance (reviewed by [33, 53]), underlining the complex etiologic nature of PD.

Driven by the neuropathology and genetics, the neurotoxicity of AS has been a major area of research in PD towards the elucidation of disease-associated mechanisms and discovery of novel therapies. Based on studies in animal models and cell cultures, including neuronal cultures, substantial evidence implicates AS aggregation in triggering different alterations including synaptic dysfunction, calcium dyshomeostasis, mitochondrial impairment, endoplasmic reticulum (ER) stress, defective autophagy, neuroinflammation, and oxidative stress [27, 39, 59, 71]. In a broader perspective, a pathological role for dysregulation of some of these cellular mechanisms is also supported by the discovery of other genetic factors causing PD. For instance, autosomal-dominant mutations in leucine-rich repeat kinase 2 (*LRRK2*), which account for the most common cause of inherited PD [53], are associated with defective autophagy and mitochondrial dysfunction [68]. Similarly, mutations in *PARK2* (Parkin, an E3 ubiquitin ligase), *PINK1* (PTEN-induced putative kinase 1) and *PARK7* (DJ-1, a protein deglycase), which are associated with early onset (age less than 40 years) PD [33, 53], directly or indirectly affect mitochondrial function either by regulating mitophagy (Parkin and PINK1) or protecting mitochondria from oxidative stress (DJ-1) [5, 59]. Some studies have also reported that mitochondrial complex I protein expression and/or activity is reduced in PD substantia nigra [29, 60] and platelets [21]. Additionally, cultures of induced pluripotent stem cells (iPSCs) derived from PD patients show defects in oxygen consumption and mitochondrial function [3, 56]. Furthermore, exposure to several chemical toxins that inhibit complex I is well documented to induce dopaminergic neuron degeneration and a parkinsonian phenotype in humans (e.g., 1-methyl-4-phenyl-1,2,3,6-tetrahydropyridine, MPTP) and in animals (e.g., MPTP, rotenone, paraquat etc.) [33, 59].

The eukaryotic elongation factor-2 kinase (eEF2K), also known as calcium/calmodulin dependent kinase III, is an important regulatory molecule in cellular protein synthesis and also in diverse forms of synaptic plasticity [23]. Upon activation, eEF2K phosphorylates its major known substrate, the eukaryotic elongation factor-2 (eEF2), on threonine-56 (Thr56), thus leading to the dissociation of eEF2 from ribosomes and stalling of mRNA translation during the elongation phase [34, 57]. eEF2K activity is increased under condition of nutrient stress via the energy sensor AMP-activated kinase (AMPK), which positively regulates eEF2K activity by phosphorylation on serine residue 398 [34, 42]. We and others have observed increased eEF2K expression and/or activity in AD post-mortem brains [28, 43, 46], and in the brains of transgenic AD mice [28, 46]. We have also shown that eEF2K inhibition prevents the toxicity of amyloid-β (Aβ) oligomers in neuronal cultures by activating the NRF2 antioxidant response, and attenuates human Aβ-induced deficits in neuronal function in *C. elegans* [28].

Mitochondrial defects (directly or indirectly associated with the aggregation of AS protein) and oxidative stress are implicated in PD pathogenesis [5, 59], and eEF2K inhibition reduces reactive oxygen species (ROS) levels in cells [10, 28]. Therefore, we hypothesized that eEF2K inhibition may mitigate AS induced neurotoxicity by reducing oxidative stress. To test this hypothesis, we first examined markers of eEF2K activity,^1^ i.e., phosphoryaltion of eEF2 on serine residue 56, in postmortem PD brains in order to establish its relevance to human pathology, and subsequent to the induction of AS pathology in transgenic mouse M83 line expressing PD-associated mutant *Ala53Thr* (A53T) AS [20, 58]. Then, we probed the effects of eEF2K inhibition on cytotoxicity, mitochondrial function and oxidative stress in AS overexpressing dopaminergic N2A cells, and on dopaminergic neuronal function in *C. elegans* expressing mutant A53T AS. By using multiple experimental approaches, we elucidate the relevance of eEF2K in AS toxicity, and discuss the potential utility of eEF2K inhibition in PD and related synucleinopathies.

## Materials and methods

### Reagents and biochemical assays

Plasmids for overexpression of AS in mammalian cells were obtained under MTA from Addgene, and included human wild type AS (pHM6-alphasynuclein-WT, Addgene #40824) and human mutant A53T AS (pHM6-alphasynuclein-A53T, Addgene #40825). Additional reagents and biochemical assays employed during these studies include: pool of small interference RNAs (SiRNAs) targeting mouse eEF2K (Santa Cruz, #sc-39,012), Cell Titer Glo ATP measurement kit (Promega, #G7570), Lactate dehydrogenase (LDH) fluorometric assay (Novus Biologicals, #NBP2–54851), Seahorse Mito stress test kit (Agilent, #103015–100), 2′,7′-dichlorodihydrofluorescein diacetate (DCFDA) fluorescent ROS reagent (ThermoFisher, #D399), and MitoTracker Green fluorescent reagent for mitochondrial mass (ThermoFisher, #M7514). Biochemical assays (LDH and ATP), Seahorse assays and flow cytometry assays were performed according to manufacturer’s recommendations and are also outlined in details below.

### Immunohistochemistry (IHC) and immunofluorescence studies on postmortem human brain sections

Five-micrometer formalin-fixed paraffin embedded separate post-mortem sections from midbrain and hippocampus of control or PD patients were provided by the laboratory of IM (co-author), as approved by the University of British Columbia Ethics Committee. Anonymized brain sections from 3 control individuals and 6 clinically and pathologically confirmed PD patients were obtained at autopsy and used in these experiments (Additional file 1: Table S1).

IHC on brain sections from human tissue was performed after deparaffinization and antigen retrieval. The following antibodies were employed to stain serial tissue sections, as indicated: antibody against phospho-eEF2 (Thr56) (Novus Biologicals, #NB100–92518) [28, 42], and antibody against phospho-alpha synuclein (pSer129; EMD Millipore, #MABN826), using the alkaline phosphatise conjugated streptavidin-biotin ABC kit (Vector Labs, # AK-5000). For destaining/bleaching neuromelanin in substantia nigra in the midbrain sections, the IHC protocol was modified slightly, as described [52] . Briefly, sections mounted on slides were incubated in a 60 °C degrees oven for 30 min and then were transferred into ambient distilled water. Then, the slides were placed in 0.25% potassium permanganate solution for 5 min. Subsequently, the slides were rinsed with distilled water. This was followed by incubation in 5% oxalic acid until section became clear. A final rinse in distilled water was performed before proceeding with the normal IHC staining as described above. Sections were counterstained with hematoxylin (Vector Labs, #H-3401). High resolution panoramic images of tissue sections for p-eEF2 and p-ASyn IHC analysis were acquired using a Leica Aperio digital slide scanner. IHC staining for p-eEF2 was quantified by manual counting of the DAB (3,3′-diaminobenzidine; Vector Labs, #SK-4100) positive cells.

For the detection of p-eEF2 (T56) and p-ASyn (S129) in the same tissue section, i.e., colocalization studies, immunofluorescence labelling was performed. For this purpose, after incubation of the tissue sections with primary antibodies as in the staining protocol described above, Alexa Fluor conjugated secondary fluorescent antibodies (Alexa Fluor 488 Goat anti-Mouse IgG, Thermo Fisher # A32723 and Alexa Fluor 594 Goat anti-Rabbit IgG, Thermo Fisher # R37119) were used for the detection. Image acquisition was performed using a Nikon Eclipse TE2000 confocal microscope.

### Animal studies

#### Husbandry

Transgenic M83^+/+^ PD mice [B6; C3-Tg (Prnp-SNCA*A53T) 83Vle/J] were kindly provided by the laboratory of Benoit Gaisson at the Centre for Translation Research in Neurodegenerative Diseases, University of Florida, USA to the laboratory of PHJ (co-author). These mice express the mutant human A53T AS under the direction of the mouse prion protein promoter [20]. The mice were housed at the Aarhus University Bartholin animal facility under conditions of 12 h light/dark cycles and received ad libitum standard laboratory chow diet. All procedures were performed in accordance with National rules and the European Communities Council Directive for the care and handling of laboratory animals. Both male and female mice were used for biochemical analyses. All genotypes were determined by PCR.

#### Intramuscular injections of alpha synuclein fibrils

Fibrillar mouse AS was prepared essentially according to an established protocol [58]. Tg M83^+/+^ were bilaterally injected with recombinant mouse AS preformed fibrils (PFF) as described [58]. Briefly, 2–3 month-old mice were anesthetized with isoflurane (1–5%) inhalation and injected intramuscularly into the hindlimb biceps femoris bilaterally. The inoculum (5 μL of 2 mg/mL PFF or PBS) was injected using a 10-μL Hamilton syringe with a 25-gauge needle. Separate syringes were used for each type of inoculums (PBS or PFF) to avoid any cross-contamination. After the injection, mice were allowed to recover under close observation before being returned to their original cage.

#### Hindlimb clasping

Assessment of hindlimb clasping behaviour was performed with a modified tail suspension test [22]. Freely moving, non-anesthesized, Tg M83^+/+^ were held by the tail and lifted in air for 10 s. Severity of clasping was assessed as follows: 1) No clasping (score 0), hindlimbs were consistently spread outward and away from the abdomen; 2) Mild clasping (score 1), one hindlimb was retracted toward the abdomen for more than 50% of the time; 3) Moderate clasping (score 2), both hindlimbs were partially retracted toward the abdomen for more than 50% of the time suspended; and 4) Severe clasping (score 3), hindlimbs were entirely retracted and touching the abdomen for more than 50% of the time suspended.

#### Quantitative RT-PCR

Total RNA from the whole brain homogenates was extracted using a commercial kit (Qiagen, #74134), and cDNA was synthesized using high capacity reverse transcriptase kit (Applied Biosystems, #4368814). The following gene specific primer pairs were used in qRT-PCR: Mouse *eef2k* (forward, 5’-CGCTTTGTACCGGGGATTCT-3′; reverse, 5′- AAGGATGGTCCTCCCACAGT-3′) and Mouse *Gapdh* (forward, 5′- CCCTTAAGAGGGATGCTGCC-3′; reverse, 5’-TACGGCCAAATCCGTTCACA-3′). The data were analyzed by relative ΔΔCT quantification method using *Gapdh* CT values as internal reference in each sample.

### Cell culture

#### Midbrain organoid cultures

Midbrain organoids were generated with a modified protocol as reported previously [49], from human iPSCs essentially as described [3]. After 35 days of differentiation, RNA from snap frozen wild type or A53T mutation carrying organoids was isolated using a commercial kit (Qiagen, # 74104), and cDNA was synthesized using Applied Biosystems high capacity reverse transcriptase kit (Thermo Fisher, # 4368814). Following gene specific primer pairs were used in qRT-PCR: Human *EEF2K*, forward 5′- CCCAAGCAGGTGGACATCAT-3′ and reverse 5’-TTGCCCTCGATGTAGTGCTC-3′ and human glyceraldehyde 3-phosphate dehydrogenase (*GAPDH*), forward 5′- GACAGTCAGCCGCATCTTCT -3′ and reverse 5′- ACCAAATCCGTTGACTCCGA -3′.

#### N2A cultures

N2A neuroblastoma cells were obtained from ATCC (#CCL-131), and maintained in DMEM (4.5 g/L glucose; Gibco, #11965–084) supplemented with 1% antibiotic-antimycotic solution (Gibco, #15240062) and 10% Fetal Bovine Serum (FBS), Cells were cultured in 6-well (500, 000 cells/well) 12-well (250,000 cells/well) or 96-well (50,000 cells/well) plates. DNA plasmid transfections were performed using Lipofectamine 2000 (Invitrogen, #11668019), and Lipofectamine RNAiMAX (Invitroge, #13778150) for siRNAs, according to the recommended procedures. After 24 h, cells were briefly washed with phosphate-buffer saline (PBS) and allowed to differentiate into neurons in a modified culture medium [59] containing DMEM (Gibco, #21969035) supplemented with 500 μM L-glutamine, 1% antibiotic-antimycotic, 2% FBS and 500 μM Dibutyryladenosine 3′,5′-cyclic monophosphate (db cAMP; Sigma, #D0627) [28, 63]. Unless indicated otherwise, differentiated N2A cells which were mock transfected, or transfected with AS plasmids (ASyn-WT or ASyn-A53T; Addgene plasmid #40824 and #40825 respectively) +/− eEF2K kd, were used in the various assays described below after 72–76 h post-transfection.

#### Cytotoxicity assays

For the lactate dehydrogenase (LDH) release, 50 μl of culture medium was collected from each well into sterile tubes and cell debris was removed by centrifugation (1100 rpm, 10 min; 4 °C) in a tabletop centrifuge. Then, 5 μl of the supernatant were carefully transferred into a 96-well black microplate cooled and kept on ice. Then, the assay reagents, as recommended by the manufacturer were added to the wells. Fluorescence (Ex/Em = λ535/λ587 nm) was measured in a Tecan microplate reader equipped with necessary filters at room temperature. Measurements were acquired in a kinetics mode every minute, after 5 s of gentle shaking, over 20 min. Stabilized fluorescence signal from each sample was collected and analyzed. For the propidium iodide (PI) cell death detection assay, the cells were gently trypsinized (0.05% Trypsin-EDTA; Gibco, #25300054), centrifuged (1100 rpm, 5 min, 4 °C) and resuspended in 500 μl of sterile ice-cold PBS containing 20% FBS and 0.001% PI (ThermoFisher, #P3566). After transferring into FACS tubes, on ice, the cells were analyzed on a FACSCalibur-Tangerine flow cytometry instrument, as described [28]. Cellular ATP levels were measured using a bioluminescence firefly luciferase assay (Cell Titer Glo, Promega) with minor modifications to the manufacturer’s instructions. Briefly, the cells were resuspended in the assay mix by thorough pipetting and transferred into a 96-well white assay plate, previously cooled on ice. Then luminescence signal was measured in a Tecan microplate reader at room temperature. Measurements were acquired in a kinetics mode every 3 min, after 5 s of gentle shaking, over 30 min. Stabilized luminescence signal from each sample was collected and analyzed.

#### Mitochondrial respiration and cellular mitochondrial content

Cellular oxygen consumption rate (OCR) was measured using the Seahorse Mito stress kit according to the supplier’s instructions. Apart from the basal OCR, a combination of pharamcological agents (components of the Seahorse Mito Stress test) enables the assessment of different aspects of cellular respiration. These include non-mitochondrial (NM) respiration, maximal respiration (MR) and spare respiratory capacity (SRC). Optimal cell density and concentrations of drugs for the assay were established according to the kit instructions/parameters. Then, 30,000 cells/well were seeded in a 96-well microplate (included in the kit) and transfections were carried out (Mock, ASyn-WT or ASyn-A53, all ± eEF2K siRNA). OCR measurements were performed in a Seahorse XF analyzer according to the assay guidelines. Basal OCR was measured over 20 min (4 cycles, 5 min/cycle), followed by exposure to oligomycin, ATP synthase inhibitor (2 μM), carbonilcyanide p-triflouromethoxyphenylhydrazone-FCCP, oxidative phosphorylation uncoupler (0.5 μM) and rotenone/antimycin, complex I and III inhibitor respectively (0.5 μM). After injection with each drug, OCR was measured over 15 min (4 cycles, 5 min/cycle). After the assay completion, the cells were gently rinsed with PBS and homogenized by pipetting in ice cold 50 μl RIPA lysis buffer (25 mM Tris-HCl pH 7.6, 150 mM NaCl, 1% NP-40, 1% sodium deoxycholate, 0.1% SDS, protease inhibitors and phosphatase inhibitors cocktail). Then, the cell lysate was transferred into microtubes, centrifuged (10,000 rpm, 10 min, 4 °C) and 25 μl of supernatant was transferred into a 96-well assay plate. Total protein in samples was determined by BCA protein assay (Pierce, #23225). OCR data was normalized to the protein content/well.

Mitochondrial content (mass) in differentiated N2A cells ± eEF2K kd was determined by labelling with Mitotracker fluorescent dye, and by quantification of mitochondrial DNA copy number. For the Mitotracker assay, cells were incubated with 50 nM Mitotracker Green FM reagent for 30 min in fresh medium. The cells were trypsinized, and centrifuged as described under the PI assay, and resuspended in 500 μl of sterile ice-cold PBS containing 20% FBS. Control cells without Mitotracker dye treatment were used as the background fluorescence signal. Separately, mitochondrial DNA (mtDNA) quantification was carried out by RT-PCR as described [47]. Briefly, nuclear DNA and mtDNA were isolated from differentiated N2A cells ± eEF2K using a Qiagen All Prep kit (#80204). Isolated samples were sonicated, then diluted to contain either 10 ng or 1 ng of DNA. This was used as an internal control to ensure that the ratio of mtDNA to nuclear DNA remained constant at different concentrations. qPCR was run on a Quant Studio 6 instrument using Fast SYBR Green Master Mix (ThermoFisher, #4309155). The primer sequences used for qPCR are as follows: mouse mitochondrial marker- *mMito* (forward, 5’-CTAGAAACCCCGAAACCAAA-3′; reverse, 5’-CCAGCTATCACCAAGCTCGT-3′) and mouse beta-2-microglobulin- *mB2M* (forward, 5’-ATGGGAAGCCGAACATACTG-3′; reverse: 5’-CAGTCTCAGTGGGGGTGAAT-3′). In each sample, mtDNA was quantified as a ratio of mtDNA to nuclear DNA (mtDNA/N) and were expressed as mtDNA copy numbers.

#### ROS measurements

For ROS detection, cells were incubated with 5 μg/mL 2, 7-dichlorofluorescein diacetate- DCFDA for 30 min in fresh medium. The cells were gently trypsinized (0.05% Trypsin-EDTA), centrifuged (1100 rpm, 5 min, 4 °C) and resuspended in 500 μl of sterile ice-cold PBS containing 20% FBS and 0.001% PI. After transferring into FACS tubes, on ice, the cells were analyzed on a FACSCalibur-Tangerine flow cytometry instrument as described previously [28]. During the analysis, dead cells were excluded from analysis based on PI staining. Control cells without DCDFA treatment were used as the background fluorescence signal.

### Western blotting

Whole brain tissue homogenates from euthanized M83^+/+^ mice were prepared in RIPA buffer (25 mM Tris-HCl pH 7.6, 150 mM NaCl, 1% NP-40, 1% sodium deoxycholate, 0.1% SDS, protease inhibitors and phosphatase inhibitors cocktail). For cellular assays, the cells were washed (ice cold PBS, 2–3 times) and lysed in RIPA buffer. Then, the mouse brain homogenates or cell lysates were briefly sonicated on ice and centrifuged (12,000 rpm, 15 min, 4 °C). Supernatant was collected and protein quantitation was done using BCA protein assay (Pierce, #23225). Then 25–40 μg of total proteins per sample were electrophoresed on 8% or 10% Bis-Tris acrylamide gels. Proteins were transferred onto a nitrocellulose membrane, incubated in blocking buffer (Li-Cor, #927–50,100]), and probed with following primary antibodies: eEF2K (Abcam, #46787), p-eEF2 Thr56 (Cell Signalling, #2331), eEF2 (Cell Signalling, #2332), ASyn (Santa Cruz #sc-12,767), p-ASyn Ser129 (Abcam, #168381, MJF-R13), and GAPDH (Cell Signaling 2118). Detection was performed using goat anti-mouse (Li-Cor, #925–32,210) or goat anti-rabbit (Li-Cor, #925–68,071) secondary antibodies conjugated with fluorescent infrared dyes using an Odyssey scanner (Li-Cor). Densitometry analysis was performed using ImageJ (NIH) [28].

### *C. elegans* studies

#### Nematode strains and culture methods

*C. elegans* strains N2 wild-type (referred to as WT-N2), RB2588 *efk-1(ok3609)*, [28] (referred to as efk-1_del_), JVR107 *Pdat-1::a-synuclein[A53T]*, [12] ((referred to as ASyn (A53T)), and STE120 *efk-1(ok3609); Pdat-1::a-synuclein[A53T]* ((generated herein, referred to as ASyn (A53T)/efk-1_del_)) were grown on Nematode Growth Medium (NGM) lite plates at 20 °C and with *E. coli* OP50 as food source, as described [28]. We used standard sodium hypochlorite bleaching and L1 stage starvation to generate synchronized populations, which were then allowed to grow for 72 h, i.e. until day two of adulthood; all assays were performed at that stage.

### Dopamine-dependent behaviour assays

#### Ethanol avoidance assay

Ethanol avoidance assays were done as described [12], on an unseeded 15 mm × 60 mm plate divided into four quadrants with a circle of 1 cm in diameter in between. Then, 1cm^3^ agarose chunks were soaked in ice-cold ethanol overnight, and placed 0.5 cm from the edge of the plate in the centre of two opposing quadrants, while the other two quadrants remained untreated. Ethanol was allowed to diffuse in media for 2 h. Actively growing day 2 old adult worms were washed five times with M9 buffer, and 150–200 worms were placed in the centre of the plate, and allowed to move for 1 h at 20 °C. Then, worms were counted manually and ethanol avoidance was calculated as [(number of worms in control quadrants) − (number of worms in ethanol quadrants))/(total number of worms)]. Three assay plates were used for each strain, and a minimum of three biological replicates was performed.

#### Pharyngeal pumping assay

The pharyngeal pumping rate was measured by manually counting the number of pumps made by each worm on seeded plate for 30 s using a Leica M205FA microscope. A total of 10–15 worms were used for each replicate. The assay was repeated at least three times on separate worms.

#### Area-restricted searching assay

Area-restricted searching assays were done as described [13]. Worms were washed very quickly to remove bacteria, and 10–15 worms were placed on an unseeded plate. To record the turning frequency, worms were videotaped at intervals of 5 and 30 min for one minute using a MoticamX camera mounted on a Leica M205FA microscope. Videotapes were analyzed manually to count the number of high-angled turns, i.e. those that exceeded 90°, including reversals and omega turns. The area-restricted searching ratio was calculated as [(number of turns/worm at 5 min)/(number of turns per worm at 30 min)]. A minimum of three replicates was performed for this assay.

### Statistics

The data were analyzed in Graphpad Prism software (version 5) or Microsoft Excel 2010, and graphs were made in Microsoft Excel 2010. Statistical differences between two sets of data were calculated by Mann-Whitney nonparametric test or unpaired T-test, as indicated in the figure legends. Multiple column datasets were analyzed by One-way ANOVA followed by Bonferonni *posthoc* analysis. Longitudinal analysis was performed by Two-way ANOVA.

## Results

### eEF2K expression and activity are increased in PD brain

**Fig. 1:**
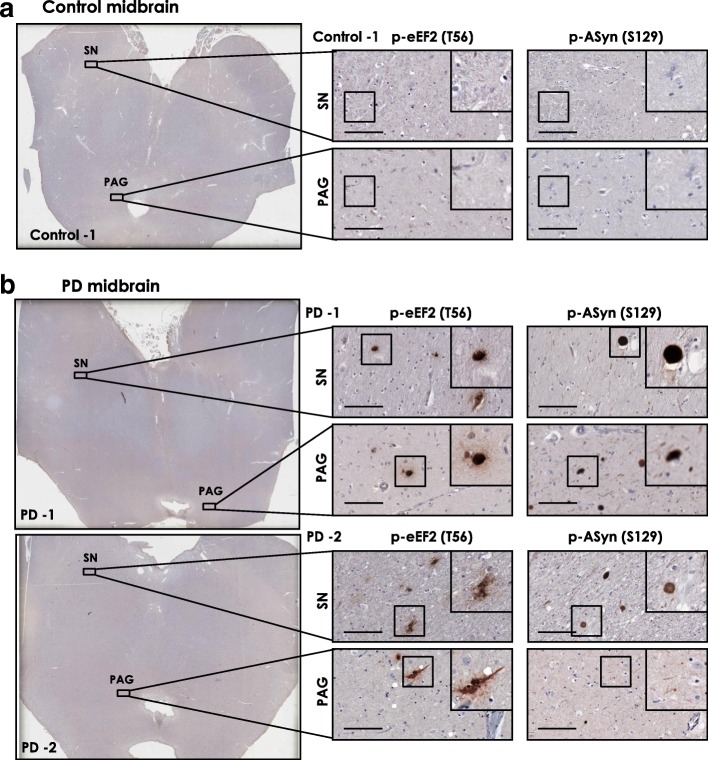
Immunostaining for phospho-eEF2 (p-eEF2, Thr56) and phospho-AS (p-ASyn, Ser129) in postmortem control and PD midbrain serial sections. **a**-**b** p-eEF2 (T56) and p-ASyn (S129) IHC in postmortem midbrain serial sections from one control (**a**) and two PD cases (**b**). IHC staining for p-eEF2 is predominantly seen in neurons in both cases, and possibly glial cells in substantia nigra in PD-2. Substantia nigra in both cases shows involvement by lewy body-LB pathology (p-ASyn, S129); while in periaqueductal gray matter, LB inclusions are seen in PD-1 and some lewy neurites are seen in PD-2. Additional control and PD midbrain IHC data are presented in Additional file 1: Figure S2-S3, and case details are included in Additional file 1: Table S1. (SN- substantia nigra; PAG- periaqueductal gray matter; scale bar, 100 μm; insets show 40× magnified view in each image)

Using postmortem brain sections from midbrain and hippocampus of controls and PD patients (Additional file 1: Table S1), we performed immunohistochemistry (IHC) analysis of eEF2 phosphorylation on threonine residue 56 (p-eEF2, T56), which reflects eEF2K activity [42, 57]. In parallel, using serial sections, we also assessed the phosphorylation of AS on serine residue 129 (p-ASyn, S129) by IHC, as it is a robust marker for AS Lewy pathology (~ 90% S129 phosphorylated AS is found in inclusions) [2, 65]. To avoid ambiguity with neuromelanin pigment found in dopaminergic neurons in substantia nigra (SN) with DAB IHC staining, we employed a modified IHC protocol [52] in order to effectively destain/bleache neuromelanin in midbrain sections without adversely affecting p-eEF2 (T56) IHC staining (Additional file 1: Figure S1a-b). Our data show that p-eEF2 (T56) immunostaining is increased in SN and periaqueductal gray (PAG) matter (gray matter surrounding cerebral aqueduct) in PD midbrain sections compared to controls (Fig. 1a-b; additional controls shown in Additional file 1: Figure S2a; additional PD cases are shown in Additional file 1: Figure S3a; quantitation of p-eEF2 IHC staining is presented in Fig. 3a). We observed that p-eEF2 IHC staining was predominantly in neurons in SN and PAG in PD cases, and in some glial cells in SN (for instance in PD-2, Fig. 1b; PD-3 and PD-4, Additional file 1: Figure S3a). As expected, we observed Lewy body pathology (p-ASyn, S129) characteristic of PD in both of these midbrain regions in PD cases but not in controls (Fig. 1a-b; Additional file 1: Figure S2a, S3a). Accordingly, Lewy body inclusion pathology was seen in most PD cases both in SN and PAG, with some lewy neurites in SN (PD-2, Fig. 1b; PD-6, Additional file 1: Figure S3a) and PAG area (PD-2, Fig. 1b; PD-3 and PD-5, Additional file 1: Figure S3a). Then, by using immunofluorescence, we assessed whether p-eEF2 (T56) immunopositivity potentially colocalizes with p-ASyn (S129), or p-eEF2 (T56) is a possible component of lewy body pathology. While we observed some neurons in PD SN which were clearly positive for both p-eEF2 (T56) and p-ASyn (S129), we also found substantial p-eEF2 (T56) immunopositivity in cells without p-ASyn (S129) and vice versa (Additional file 1: Figure S4b).

**Fig. 2:**
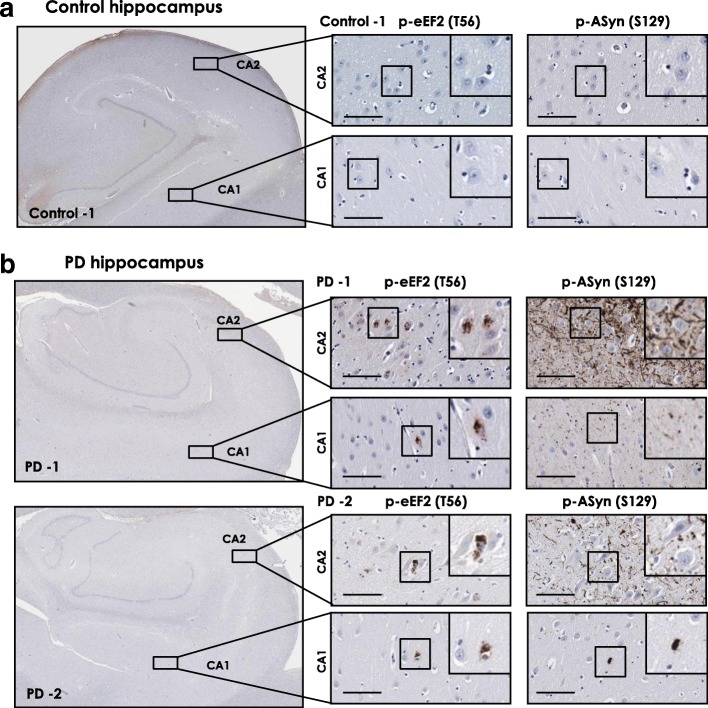
Immunostaining for phospho-eEF2 (p-eEF2, Thr56) and phospho-AS (p-ASyn, Ser129) in postmortem control and PD hippocampus serial sections. **a**-**b** p-eEF2 (T56) and p-ASyn (S129) IHC in postmortem hippocampus serial sections from one control (**a**) and two PD cases (**b**). IHC staining for p-eEF2 in PD cases is seen predominantly in CA1 and CA2 neurons. Lewy body inclusions and neurites (p-ASyn, S129) are seen in both PD cases, with pronounced involvement of CA2. IHC data concerning CA3 and dentate gyrus from PD-1 and PD-2 are included in Additional file 1: Figure S7a. Additional control and PD hippocampus IHC data are presented in Additional file 1: Figure S5-S7, and case details are included in Additional file 1: Table S1 (CA1 and CA2- hippocampal cornu ammonis fields 1 and 2 respectively; scale bar, 100 μm; insets show 40× magnified view in each image)

**Fig. 3:**
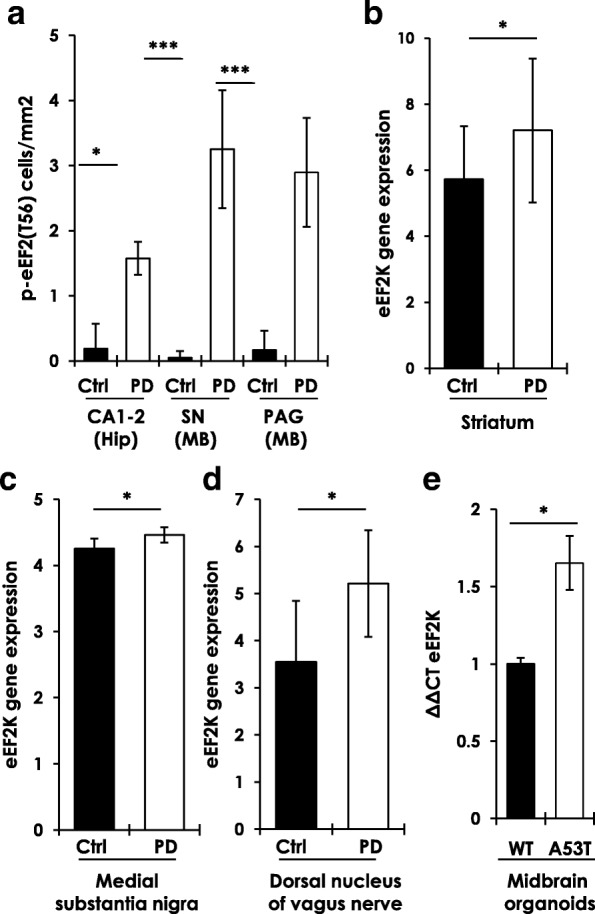
eEF2K expression and activity in PD brain. **a** Quantitation of p-eEF2 (T56) IHC (3,3′-Diaminobenzidine-DAB staining) in postmortem hippocampus- Hip (CA1 and CA2 fields) and midbrain- MB (SN-substantia nigra, and PAG-peri-aqueductal gray matter) sections from 3 control and 6 PD cases (Additional file 1: Table S1; counts from at least 6 high power fields from each control or PD section; Mann–Whitney test, *p < 0.05, ***p < 0.005; error bars indicate Mean ± S.D.). **b**-**d** eEF2K mRNA expression in control and PD striatum (**b**), medial substantia nigra (**b**) and dorsal nucleus of vagus nerve (**d**). The following publicly available transcriptomic profile datasets were analyzed on the National Center for Biotechnology Information (NCBI) Gene Expression Omnibus (GEO) platform: Striatum (**b**)- dataset GEO accession # GSE28894, Illumina human Ref-8 v2.0 expression beadchip platform, probe ID ILMN_1789171, controls *n* = 15 and PD *n* = 15; Medial substantia nigra (**c**)- dataset GEO accession # GSE8397, Affymetrix Human Genome U133B Array, probe ID 225546_at, controls *n* = 8 and PD *n* = 15; Dorsal nucleus of vagus (**d**)- dataset GEO accession # GSE43490, Agilent-014850 Whole Human Genome Microarray, probe ID A_24_P716162, controls *n* = 6 and PD *n* = 7. (Mann–Whitney test, *p < 0.05; error bars in 3b-d indicate Mean ± S.D.). **e** Relative eEF2K mRNA expression in human iPSCs derived cultured midbrain control (WT, *n* = 3) or A53T (*n* = 2) mutation carrying organoids (T-test, *p < 0.05; error bars indicate Mean ± S.D.)

Previous reports, including our own published data, show that phosphorylation of eEF2 (p-eEF2, T56) is strongly increased in postmortem hippocampus and mesial temporal cortex in AD, the major neurodegenerative disease with dementia [28, 43, 46]. Among the PD cases examined here, PD-1 and PD-5 were also clinically diagnosed with PD with dementia (PDD), which is usually seen in longstanding PD [1, 2]. Therefore, we assessed p-eEF2 (T56) in postmortem hippocampus sections from control and PD cases. We found increased p-eEF2 IHC staining in hippocampal CA1 and CA2 (CA, *cornu ammonis*) fields in PD cases compared to controls, predominantly in neurons (Fig. 2a-b; additional controls shown in Additional file 1: Figure S5a-b; additional PD cases shown in Additional file 1: Figure S6a, panoramic views; Additional file 1: Figure S7a-b, magnified field views; quantitation of p-eEF2 IHC staining in areas CA1-CA2 is presented in Fig. 3a). There was little or none p-eEF2 immunopositivity in CA3 and dentate gyrus (DG) in all PD cases, except PD-1 and PD-3 (CA3, Additional file 1: Figure S7a-b). We also assessed Lewy body pathology in hippocampal sections from these control and PD cases, since Lewy pathology in hippocampus is found at advanced neuropathological stages of PD (Braak PD staging, stages 4–6) [1, 36]. Our IHC analysis for p-ASyn (S129) showed varying degrees of Lewy body inclusions (PD-1 and PD-2, Fig. 2b; PD-3 and PD-6, Additional file 1: Figure S7b) and Lewy neurites pathology (PD-1 and PD-2, Fig. 2b; PD-6, Additional file 1: Figure S7b), with pronounced involvement of hippocampal CA2 field in most PD cases (Fig. 2a-b and Additional file 1: Figure S7a-b).

We also queried multiple transcriptome datasets publicly available in the National Center for Biotechnology Information (NCBI) Gene Expression Omnibus (GEO) platform for eEF2K mRNA expression in PD brain. Significantly increased eEF2K mRNA expression was found in Striatum (Fig. 3b, GEO accession # GSE28894), medial substantia nigra (Fig. 3c, GEO accession # GSE8397), and dorsal nucleus of vagus (dmX) (Fig. 3d, GEO accession # GSE43490). Collectively, these data provide strong evidence for aberrant eEF2K expression and activity in PD brain. Finally, we also performed quantitative PCR in cultured midbrain organoids derived from human iPSCs [3, 49], and found significantly increased eEF2K mRNA expression in A53T mutant AS carrying organoids compared to wild type controls (Fig. 3e).

### eEF2K expression and activity are increased in M83^+/+^ transgenic PD mouse brains subsequent to induction of AS neuropathology

**Fig. 4:**
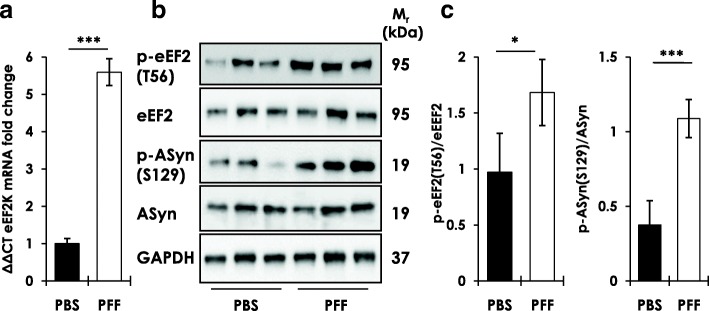
Brain eEF2K expression and activity in transgenic M83^+/+^ PD mice. **a** eEF2K mRNA levels in whole brain homogenates from transgenic M83^+/+^ PD mice intramuscularly (IM) injected bilaterally with phosphate buffered saline (PBS, *n* = 10) or pre-formed fibrillar (PFF, *n* = 13) mouse wild type AS. (Mann–Whitney test, ***p < 0.005; error bars indicate Mean ± S.D.). **b**-**c** Western blot analysis of p-eEF2 (T56) and p-ASyn (S129) in whole brain homogenates from transgenic M83^+/+^ PD mice intramuscularly (IM) injected bilaterally with phosphate buffered saline (PBS) or pre-formed fibrillar (PFF) mouse wild type AS (**b**), and corresponding densitometry analysis (**c**) (*n* = 7/group; Mann–Whitney test, *p < 0.05, ***p < 0.005; error bars indicate Mean ± S.D.)

To further establish the relevance of eEF2K to AS-related pathology in PD, we analyzed brain eEF2K expression in transgenic PD M83^+/+^ mice, subsequent to induction of AS pathology by intramuscular injection of pre-formed fibrillar PFF AS [20, 58]. Within 8–10 weeks post-injection, the PFF AS injected M83^+/+^ mice show profound motor neuron loss, AS inclusions, progressive motor deficits and reduced survival at much earlier ages than native M83^+/+^ mice [58]. For these analyses, PFF AS injected M83^+/+^ mice (injected at 2–3 months of age) were used after 8–10 weeks post-injection when typical motor abnormalities such as hindlimb paralysis (Additional file 1: Figure S8a) and severe hindlimb clasping behaviour (Additional file 1: Figure S8b-c) were evident. Our data show that compared to PBS injected M83^+/+^ mice, brain eEF2K mRNA expression is remarkably increased (~ 5–6 fold) in PFF AS injected M83^+/+^ moribund (clasping score 3, see Materials and Methods) mice (Fig. 4a). Accordingly, we also found increased eEF2K activity (p-eEF2, T56) and induction of pathological AS phosphorylation (p-ASyn, S129) by western blotting in PFF AS injected M83^+/+^ (Fig. 4b-c). These data further support our hypothesis regarding a role of eEF2K in AS neurotoxicity.

### AS overexpression increases eEF2K activity, while eEF2K inhibition reduces AS cytotoxicity in dopaminergic N2A cells

As mentioned above, AS-induced neurotoxicity is considered to play an important role in neurodegeneration in PD [39, 71]. Indeed, overexpression of AS in cultured cells promotes AS aggregation, increases oxidative stress, and reduces cell survival [4, 8]. This has been observed for the wild-type and mutant forms of AS, including the A53T mutant AS, which accelerates AS aggregation and pathology [39, 41]. To study the effects of eEF2K inhibition on AS toxicity, we employed differentiated mouse neuroblastoma N2A cells overexpressing either wild-type AS (ASyn-WT) or the A53T mutant (ASyn-A53T) +/− siRNA mediated eEF2K knockdown (kd), and measured cytotoxicity in these cells. Differentiated N2A cells exhibit many features of mature dopaminergic neurons including functional neurotransmitter receptors [63], and are widely used to study the toxicity of amyloid proteins [14, 28].

**Fig. 5:**
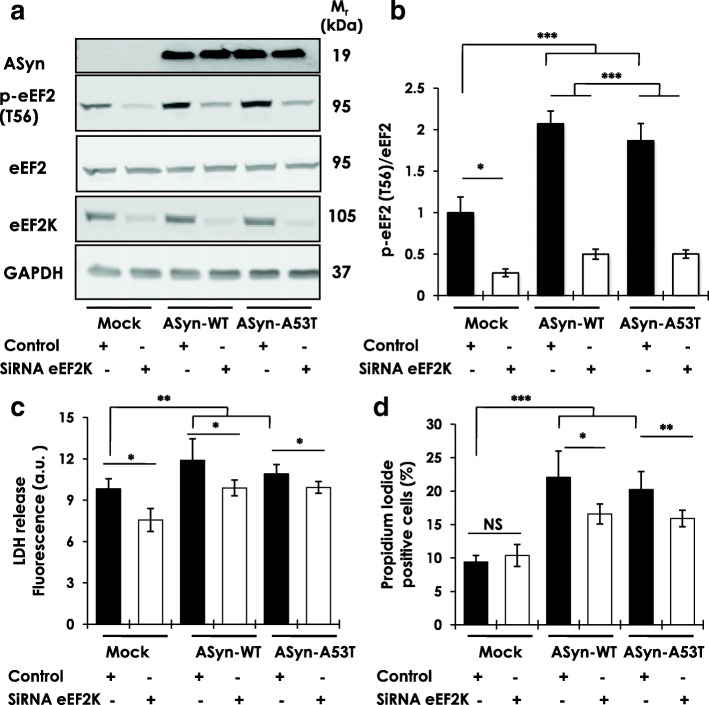
Effects of eEF2K inhibition on human AS cytotoxicity in differentiated N2A cells. **a**-**b** Western blot analysis of p-eEF2 (T56) levels in N2A cells subsequent to transient overexpression of human wild type or mutant A53T AS, with or without siRNA mediated eEF2K knockdown (**a**), and corresponding densitometry analysis (**b**) (*n* = 6–9/group from three independent experiments; One-way ANOVA *post-hoc* Bonferroni test, *p < 0.05, ***p < 0.005; error bars indicate Mean ± S.E.M). c Measurements of cytotoxicity by lactate dehydrogenase-LDH release in the culture medium (**c**) and FACS analysis of propidium iodide-PI staining (**d**) in N2A cells subsequent to transient overexpression of human wild type or mutant A53T AS, with or without siRNA mediated eEF2K knockdown (*n* = 9–12/group from three independent experiments; One-way ANOVA *post-hoc* Bonferroni test, *p < 0.05, **p < 0.01, ***p < 0.005, NS = not significant; error bars indicate Mean ± S.D.)

Overexpression of ASyn-WT or ASyn-A53T increased p-eEF2 (T56) levels in N2A cells, which was reduced by eEF2K kd (Fig. 5a-b). We then assessed AS cytotoxicity by measuring the activity of lactate dehyrogenase (LDH) in the culture medium. LDH is a cytoplasmic enzyme released under conditions of cell membrane damage and during toxic stress in neuronal cultures [37]. As expected, overexpression of ASyn-WT or ASyn-A53T led to increased LDH release (72 h post-transfection), which was reduced significantly by eEF2K kd in both ASyn-WT or ASyn-A53T expressing cells (Fig. 5c). Next, we measured cytotoxicity in these cultures by labelling with propidium iodide (PI), another cell permeable marker of cell death. Overexpression of ASyn-WT or ASyn-A53T resulted in increased cell death (72 h post-transfection), as measured by flow cytometry analysis of PI staining, and eEF2K kd significantly improved viability in these cultures (Fig. 5d).

### eEF2K inhibition mitigates AS induced mitochondrial dysfunction and oxidative stress in N2A cells

Next, we assessed whether the cytoprotective effects of eEF2K inhibition against AS toxicity are mediated by changes in mitochondrial function, since AS inhibits mitochondrial respiration and complex I activity [8, 55]. First, we characterized cellular respiration (oxygen consumption rate, OCR) in differentiated N2A cells following eEF2K kd without ASyn overexpression. This is important since eEF2K regulates highly energy consuming process of elongation during mRNA translation, and we wanted to assess if possible metabolic reprogramming in cells due to eEF2K inhibition does not impair mitochondrial function [15, 34]. Intriguingly, N2A cells with eEF2K kd exhibited significantly higher OCR under basal conditions, and maximal respiration subsequent to the treatment with FCCP (uncoupler of oxidative phosphorylation) than control cells (Additional file 1: Figure S9a). To investigate if the increased respiration in eEF2K kd cells under basal conditions is linked to an increase in the mitochondrial mass, we quantified mitochondrial content in control and eEF2K kd cells. However, there were no significant differences in mitochondrial mass by flow cytometry analysis using the fluorescent Mitotracker reagent (Additional file 1: Figure S9b), or by mitochondrial mtDNA quantification (Additional file 1: Figure S9c). These data demonstrate healthy mitochondrial function in eEF2K kd cells, and suggest that, compared to control cells, cellular respiration in eEF2K kd cells is increased predominantly due to enhanced mitochondrial respiration (Additional file 1: Figure S9a; compare changes in basal respiration and maximal respiration in control vs. eEF2K kd cells) without significant changes in mitochondrial content (Additional file 1: Figure S9b-c).

**Fig. 6:**
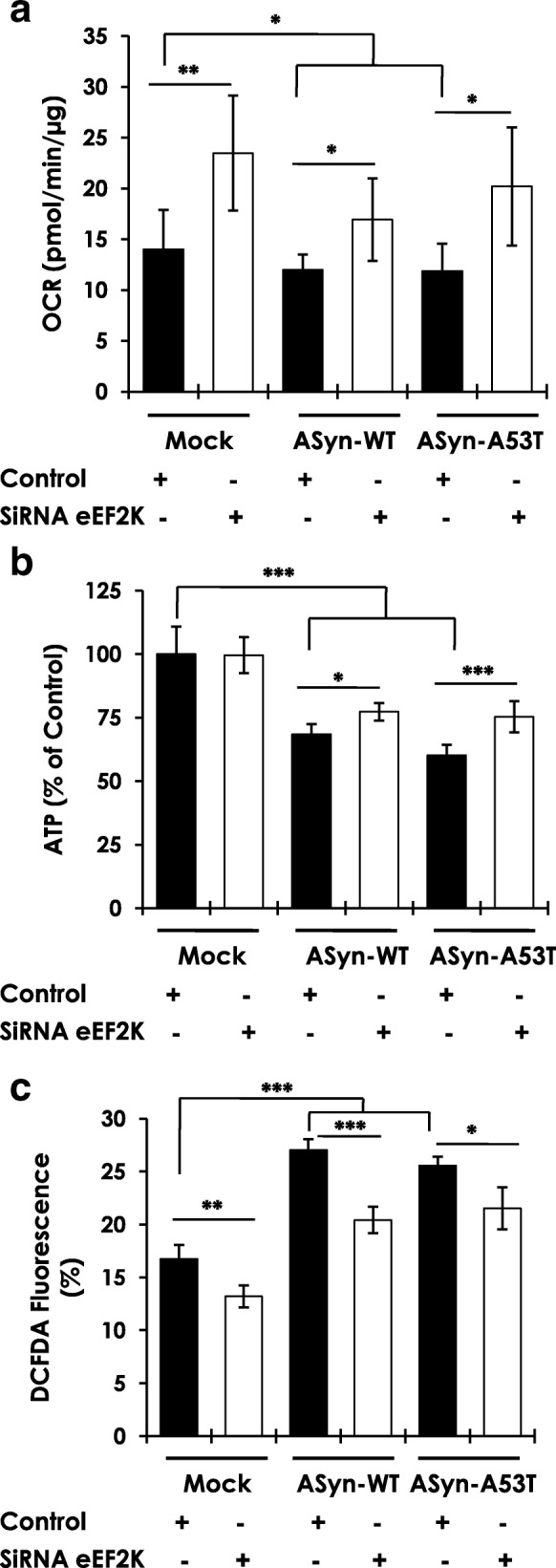
Effects of eEF2K inhibition on mitochondrial dysfunction and oxidative stress induced by human AS in differentiated N2A cells. **a**-**b** Measurements of basal oxygen consumption rate-OCR (**b**) and ATP levels (**c**) in N2A cells subsequent to transient overexpression of human wild type or mutant A53T AS, with or without siRNA mediated eEF2K knockdown (*n* = 9–12/group from three independent experiments; Unpaired T-test, *p < 0.05, **p < 0.01, ***p < 0.005; error bars indicate Mean ± S.D.). **c** Flow cytometry analysis of reactive oxygen species (ROS), measured by DCFDA staining, in N2A cells subsequent to transient overexpression of human wild type or mutant A53T AS, with or without siRNA mediated eEF2K knockdown (*n* = 9/group from three independent experiments; Unpaired T-test, *p < 0.05, **p < 0.01, ***p < 0.005; error bars indicate Mean ± S.D.)

Having established that eEF2K kd per se does not negatively affect mitochondrial function in N2A cells, we proceeded to assess the effects of eEF2K kd on AS induced mitochondrial dysfunction [8, 27]. We investigated this activity in differentiated N2A cells with overexpression of ASyn-WT or ASyn-A53T +/− eEF2K kd (72 h post-transfection). There was a noticeable reduction in basal OCR in cells overexpressing ASyn-WT, or ASyn-A53T compared to mock transfected cells (Fig. 6a). eEF2K kd led to significant improvement of the OCR under all conditions (compare Mock control vs. Mock+sieEF2K, ASyn-WT vs. ASyn-WT + sieEF2K and ASyn-A53T vs. ASyn-A53T + sieEF2K; Fig. 6a). Then, we measured cellular ATP levels under identical conditions, and found that overexpression of ASyn-WT or ASyn-A53T significantly reduced cellular ATP content reflecting AS toxicity (Fig. 6b). While eEF2K kd had negligible effect on ATP content in mock transfected cells, it attenuated the loss of ATP in ASyn overexpressing cells (compare ASyn-WT vs. ASyn-WT + sieEF2K and ASyn-A53T vs. ASyn-A53T + sieEF2K; Fig. 6b). Together, these data suggest that transient AS (WT or A53T) overexpression is associated with mitochondrial dysfunction in these dopaminergic cultures, which is rescued by eEF2K kd.

Mitochondrial dysfunction, including complex I inhibition, is associated with increased production of reactive oxygen species (ROS) and oxidative stress in cells [44]. As mentioned earlier, these processes, i.e., impaired mitochondrial function and increased ROS, are also implicated in AS toxicity [4]. Indeed, previous studies have shown that ROS levels are increased in AS overxpressing cells [27, 32]. Accordingly, we found that overexpression of ASyn-WT or ASyn-A53T increased ROS levels compared with mock transfected cells, as measured by flow cytometry analysis of the ROS detection reagent, DCFDA (Fig. 6c). Moreover, we found that eEF2K kd significantly reduced ROS in these cultures, in line with previously reported effects of eEF2K inhibition on cellular ROS levels [10, 28]. Collectively, these data suggest a role of eEF2K in AS toxicity (Fig. 5a-b), and demonstrate that eEF2K inhibition reduces AS toxicity by improving mitochondrial function and reducing ROS (Fig. 6a-c).

### *Deletion of* efk-1 *improves dopaminergic neuronal function in a C. elegans model of AS neurotoxicity*

To assess the in vivo impact of eEF2K inhibition as a means of improving AS-mediated neurotoxicity, we used a *C. elegans* model of PD. *C. elegans* possess four bilaterally symmetric pairs of dopaminergic neurons that are critical for adaptations to mechanosensory stimuli, and for the regulation of complex behaviours such as foraging, movement, and egg-laying [50, 69]. Accordingly, this worm model is widely used in PD research to investigate the significance of specific mutations and variations, and to screen for candidate disease-modifying small molecules [70]. We studied a previously generated *C. elegans* strain that transgenically expresses human AS A53T mutant, which results in age-related degeneration of dopaminergic neurons and in defects in dopaminergic function in these worms [12]. Using this strain, we assessed dopaminergic neuron function with or without concomitant deletion of *efk-1*, the eEF2K ortholog in worms [(ASyn (A53T) and ASyn (A53T)/efk-1_del_ strains; see Materials and Methods)]. We assessed the effects of *efk-1* deletion in A53T AS expressing worms in three behavioural responses that are considered to be mediated predominantly by dopaminergic neurons in worms: ethanol avoidance, pharyngeal pumping, and area restricted searching (see Materials and Methods). We hypothesized that, in view of the cytoprotective effects of eEF2K inhibition against AS toxicity in cultured dopaminergic N2A cells (Figs. 5-6), *efk-1* deletion would improve AS-A53T-induced dopaminergic neuron dysfunction in worms.

**Fig. 7:**
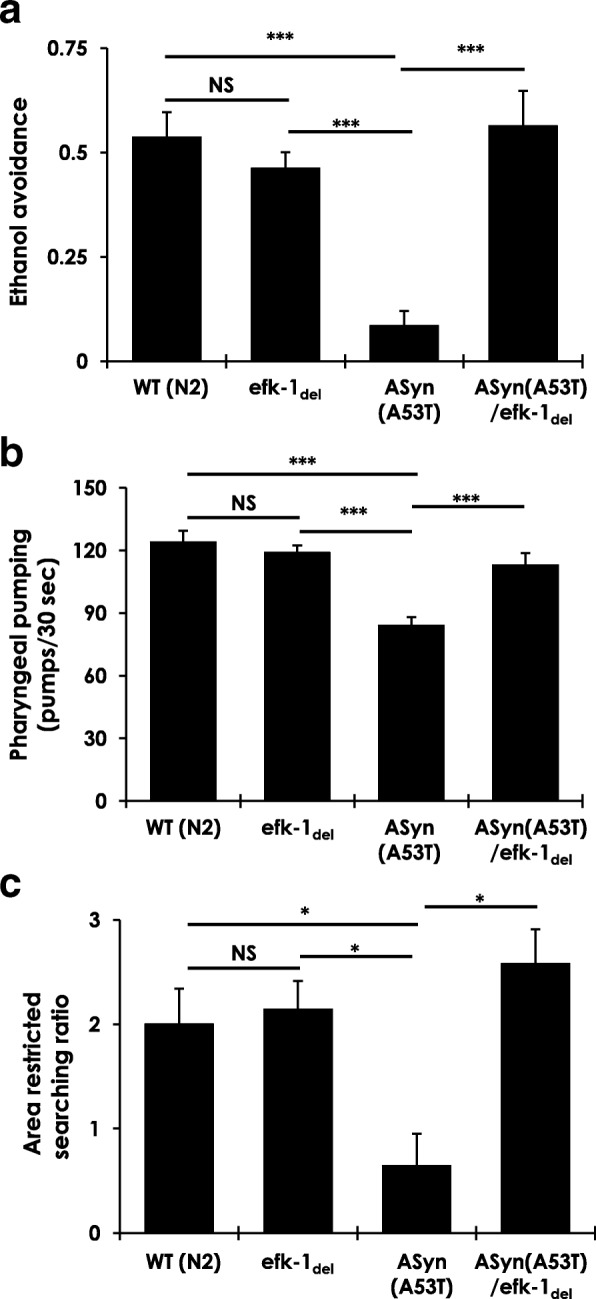
Effects of efk-1 deletion on ethanol avoidance, pharyngeal pumping, and area restricted searching behavior in *C. elegans* expressing human AS-A53T. **a**-**c** Analysis of ethanol avoidance (*n* = 100–150 worms/measurement) (**a**), pharyngeal pumping (*n* = 10–15 worms/measurement) (**b**), and area restricted searching behavior (*n* = 10–15 worms/measurement) (**c**) in control (wild type N2) worms, in *efk-1(ok3609)* null mutant worms (efk-1_del_), in worms expressing human mutant AS-A53T (*Pdat-1::a-synuclein[A53T]),* and in *efk-1(ok3609)* null mutant worms expressing AS-A53T (*efk-1(ok3609); Pdat-1::a-synuclein[A53T]*). Statistical analysis: one-way ANOVA with post-hoc Bonferroni test; *p < 0.05, ***p < 0.005, NS = not significant; error bars indicate mean ± S.E.M. from at least three independent experiments

In ethanol avoidance assays, worms exhibit an aversive response to acute ethanol exposure and this response is dependent on adequate sensory motor co-ordination [40]. Notably, *efk-1* deletion alone had no significant effects on this response (Fig. 7a; compare WT (N2) worms with efk-1_del_), while, as previously reported, expression of ASyn-A53T led to a worsened response (Fig. 7a) [12]. Importantly, loss of *efk-1* completely rescued the reduced ethanol avoidance of ASyn-A53T expressing worms [(Fig. 7a; compare ASyn (A53T) with ASyn (A53T)/efk-1_del_)]. In pharyngeal pumping assays, rhythmic contractions (pumping) of the pharynx serve as marker of neuromuscular function and rely on a complex neural integration within the autonomic activity [64]. This response is essential for feeding and generation of consequent isthmus peristalsis. As expected, we observed no difference in the pharyngeal pumping activity between wild type worms and worms lacking *efk-1* [(Fig. 7b; compare WT (N2) with efk-1_del_)]. ASyn-A53T expressing worms showed a modest, but significant, reduction in pumping activity, which was restored significantly in ASyn-A53T worms with *efk-1* deletion [(Fig. 7b; compare ASyn (A53T) with ASyn (A53T)/efk-1_del_)]. Finally, the area-restricted search behaviour reflects foraging, such that the worms show an adaptive response by reducing turning frequency to the presence of food; this response is mediated by neural circuits involving dopaminergic and glutamatergic signalling [24]. As seen with the other two assays, *efk-1* deletion alone negligibly affected the performance of worms in the area restricted search assay [(Fig. 7c; compare WT (N2) worms with efk-1_del_)], whereas ASyn-A53T mutant expressing worms showed significant defects in this assay (Fig. 7c) as reported previously [12]. Critically, this defect was completely rescued in ASyn-A53T worms lacking *efk-1* [(Fig. 7c; compare ASyn (A53T) with ASyn (A53T)/efk-1_del_)]. Taken together, *efk-1* deletion improves three independent behaviours known to rely dominantly on normal dopaminergic neuron function. These data support our hypothesis and demonstrate that *efk-1* deletion mitigates the deleterious effects of ASyn (A53T) on the function of neural circuits involving dopaminergic neurons in *C. elegans*, and further point to an in vivo role for eEF2K signaling in AS-mediated neurotoxicity.

## Discussion

Principally driven by the early genetic findings in inherited PD and PD neuropathology, a significant effort in the discovery of novel therapies in PD and other synucleinopathies has been to target AS production and/or aggregation [39, 71]. Although such studies have shown promising results in preclinical research, their translation into clinically implementable therapies has yet to be realized [71]. An additional area of research in potential therapies in PD has been to address AS neurotoxicity, and to target cellular mechanisms that potentially render neurons susceptible to AS neurotoxicity [38].

Our data suggest that eEF2K is one possible mechanism that is pathologically involved in AS-mediated toxicity, and that its inhibition represents a novel therapeutic strategy in PD. Our findings demonstrate that eEF2K activity is increased in postmortem PD midbrain (substantia nigra and periaqueductal gray matter) and in hippocampus (CA1 and CA2 regions), with the concomitant presence of Lewy pathology (phosphorylation of AS on Ser-129). Additionally, analysis of publicly available microarray datasets revealed increased eEF2K expression in striatum, medial substantia nigra and dorsal nucleus of vagus (dmX) in PD. Hence, dysregulated eEF2K expression and/or activity are observed in multiple brain regions that are affected in PD. We further show that induction of aggressive AS pathology in M83^+/+^ transgenic PD mice, by intramuscular PFF AS injection, is associated with enhanced brain eEF2K expression and activity. In addition, transient overexpression of AS (WT or A53T mutant) is associated with cytotoxicity and oxidative stress in dopaminergic N2A cells, and leads to eEF2K activation in these cultures. Moreover, eEF2K inhibition mitigated the cytotoxic effects of AS overexpression in cells and prevented the deficits in dopaminergic function in *C. elegans* due to transgenic AS-A53T expression. These observations are supported by previous reports showing that eEF2K inhibition reduces oxidative and ER stress, processes associated with AS toxicity [6, 10, 28].

It is noteworthy that brain areas showing increased eEF2K activity in PD cases examined here are part of distinct neurotransmitter networks that are affected at different neuropathological stages in PD [7, 65]. Considering the neuronal populations found in these distinct anatomical brain areas and their connectivity, it has been postulated that the clinical spectrum of PD symptoms (i.e., motor, autonomic or cognitive) may arise depending on the extent of Lewy pathology and/or cell loss [7, 33]. From a neurological perspective, it is commonly thought that the lesions in the striatum or substantia nigra underlie the motor symptoms due to neurotransmitter imbalance in the nigrostriatal system causing defective motor control and muscle tone, and this assertion is supported by the studies in animal models [18]. Furthermore, the loss of cholinergic projections from dorsal nucleus of vagus (dmX) is implicated in the autonomic dysfunction in PD [65]. The periaqueductal gray (PAG) matter is also affected with AS inclusions in PD, Dementia with Lewy bodies (DLB), and Multiple system atrophy (MSA) [61]. This cell dense region harbours many distinct neuronal populations with projections linking forebrain and lower brain stem, and in mammals is involved in autonomic control, cardiovascular function, pain modulation, wakefulness, rapid eye movement (REM) sleep and vocalization [55]. Finally, Lewy body pathology is present in hippocampus at advanced neuropathological stages in PD, and thought to underlie cognitive symptoms [7, 33, 65].

The significance of enhanced eEF2K expression and/or activity in the aforementioned brain areas in PD remains unknown. Given the critical role of eEF2K in dendritic mRNA transaltion and synaptic integrity [34], it is plausible that aberrant eEF2K activity may underlie the dysfunction in these neuronal populations. Supporting this, some studies suggest a role for eEF2K in synaptic plasticity, in particular mGluR5-mediated long-term depression [66], a mechanism purported to underlie synaptic dysfunction in AD and other neuropsychiatric conditions [11]. Furthermore, eEF2K activity is also increased upon exposure to excitatory stimuli in neuronal cultures [23, 35, 66], and in response to nutrient deprivation (by energy sensor AMP-activated kinase). However, it remains to be established whether some of the aforementioned stimuli enhancing eEF2K activity also underlie increased eEF2K activity in PD. In this regard, activity of AMP-kinase, an important regulator of eEF2K in response to metabolic stimuli, is increased in cells and/or rodents after exposure to mitochondrial toxins (e.g., MPTP, rotenone) [9, 36]. In postmortem PD brain, activated AMPK has been detected near the rim of Lewy bodies in the cytoplasm as opposed to nuclear staining in controls [30]. However, the beneficial effects of AMPK inhibition in PD models have not been conclusively established [17], and in some studies AMPK activation is protective [51]. A recent report showed aberrations in the expression of factors controlling protein synthesis (e.g., ribosomal proteins, translation initiation and elongation factors) in postrmortem PD brain, including reduced eEF2 levels in PD midbrain and cortex [19], supporting the notion of dysregulated mRNA translation in PD. Determining whether increased eEF2K activity plays a role in the synaptic defects of PD, or occurs due to aberrations within the translational machinery, represents a challenging task due to the complex nature of PD etiology and the possible involvement of multiple neurotransmitter systems as the disease progresses. For simplicity, it is plausible to suggest that increased eEF2K activity may be part of some stress-adaptive pathway, which plays a protective role in the short term under disturbed cellular homeostasis. However, chronic overactivation of this pathway in pathological states may be detrimental due to aberrations in mRNA translation and dendritic protein synthesis [28, 46].

From a therapeutic perspective, development of novel eEF2K inhibitors is actively being pursued towards novel experimental therapies in cancer due to its role in metabolic adaptations in cancer cells for survival under nutrient deprivation, including nervous system malignancies [16, 34, 42]. Here, we show that eEF2K inhibition augments mitochondrial respiration, reduces oxidative stress and prevents AS toxicity in dopaminergic N2A cells. The mechanism of increased mitochondrial function subsequent to eEF2K inhibition in dopaminergic N2A cells remains to be deciphered. We previously reported that eEF2K inhibition in dopaminergic N2A cells induces an NRF2 antioxidant response, and blocks the toxicity of Aβ oligomers in neuronal cultures [28]. NRF2 is a master regulator of cellular redox homeostasis under physiological and pathological conditions due to its ability in controlling the expression of antioxidant genes [31]. However, NRF2 is also known to regulate cellular metabolism (e.g., glutamine biogenesis), and affects mitochondrial structure and function such as ATP production, fatty acid oxidation and structural integrity [26, 48]. Accordingly, cells and mitochondria derived from NRF2 knockout mice show reduced respiration, lower ATP levels and impairments in mitochondrial fatty acid oxidation [25, 45]. Furthermore, several small molecules activators of NRF2 pathway have shown beneficial effects in restoring mitochondrial function under conditions of redox stress in cell cultures, and also in models of neurodegenerative diseases [26]. One such therapeutic molecule, dimethyl fumarate (DMF, Tecfidera), used to treat multiple sclerosis, exerts anti-inflammatory and antioxidant effects in cell culture and animal studies by activating NRF2 antioxidant response [1]. DMT administration prevents neurodegeneration in mice treated with MPTP [71], a mitochondrial toxin associated with chemically induced parkinsonism in humans and animals [1]. In this context, our findings raise the possibility that eEF2K can also be targeted in PD to mitigate AS-induced oxidative stress, and potentially neuronal dysfunction in this disease.

## Conclusion

By employing multiple experimental models, our data support the relevance of eEF2K in PD, and of eEF2K inhibition in mitigating AS-induced oxidative stress and neuronal dysfunction. We anticipate that our findings will stimulate further mechanistic studies and a careful evaluation of eEF2K inhibition in PD, and potentially other neurodegenerative diseases.

## Additional file

Additional file 1: **Table S1.** Control and PD cases; **Figure S1.** Melanin bleaching in postmortem midbrain sections and immunostaining for phospho-eEF2 (p-eEF2, Thr56); **Figure S2.** Immunostaining for phospho-eEF2 (p-eEF2, Thr56) and phospho-AS (p-ASyn, Ser129) in postmortem control midbrain sections; **Figure S3.** Immunostaining for phospho-eEF2 (p-eEF2, Thr56) and phospho-AS (p-ASyn, Ser129) in postmortem PD midbrain sections; **Figure S4.** Detection of phospho-eEF2 (p-eEF2, Thr56) and phospho-AS (p-ASyn, Ser129) in postmortem control and PD midbrain sections by immunofluorescence; **Figure S5.** Immunostaining for phospho-eEF2 (p-eEF2, Thr56) and phospho-AS (p-ASyn, Ser129) in postmortem control hippocampus sections; **Figure S6.** Immunostaining for phospho-eEF2 (p-eEF2, Thr56) in postmortem PD hippocampus sections- Panoramic views; **Figure S7.** Immunostaining for phospho-eEF2 (p-eEF2, Thr56) and phospho-AS (p-ASyn, Ser129) in postmortem PD hippocampus sections; **Figure S8.** Effects of intramuscularly injected pre-formed fibrillar (PFF) AS on motor phenotype and survival of transgenic M83+/+ PD mice and **Figure S9.** Mitochondrial respiration and mitochondrial mass in differentiated N2A cells subsequent to eEF2K knockdown. (PDF 2444 kb).

## Abbreviations

AD: Alzheimer disease
AS: Alpha synuclein
eEF2: Eukaryotic elongation factor-2
eEF2K: Eukaryotic elongation factor-2 kinase
MPTP: 1-methyl-4-phenyl-1,2,3,6-tetrahydropyridine
PD: Parkinson disease
PFF: Pre-formed fibrils
ROS: Reactive oxygen species

## Footnotes

1 The term eEF2K activity in this manuscript denotes phosphorylation of eEF2, the major known substrate for eEF2K, on serine residue 56 assessed by immunohistochemistry, immunofluorescence or western blot.
