# Supplementary material for: Activity of translation regulator eukaryotic elongation factor-2 kinase is increased in Parkinson disease brain and its inhibition reduces alpha synuclein toxicity
Source: Acta Neuropathol Commun. 2018 Jul 2;6:54. doi: 10.1186/s40478-018-0554-9 (PMC6027557; doi:10.1186/s40478-018-0554-9)
Supplement: Supplementary file 1 — Table S1. Control and PD cases; Figure S1. Melanin bleaching in postmortem midbrain sections and immunostaining for phospho-eEF2 (p-eEF2, Thr56); Figure S2. Immunostaining for phospho-eEF2 (p-eEF2, Thr56) and phospho-AS (p-ASyn, Ser129) in postmortem control midbrain sections; Figure S3. Immunostaining for phospho-eEF2 (p-eEF2, Thr56) and phospho-AS (p-ASyn, Ser129) in postmortem PD midbrain sections; Figure S4. Detection of phospho-eEF2 (p-eEF2, Thr56) and phospho-AS (p-ASyn, Ser129) in postmortem control and PD midbrain sections by immunofluorescence; Figure S5. Immunostaining for phospho-eEF2 (p-eEF2, Thr56) and phospho-AS (p-ASyn, Ser129) in postmortem control hippocampus sections; Figure S6. Immunostaining for phospho-eEF2 (p-eEF2, Thr56) in postmortem PD hippocampus sections- Panoramic views; Figure S7. Immunostaining for phospho-eEF2 (p-eEF2, Thr56) and phospho-AS (p-ASyn, Ser129) in postmortem PD hippocampus sections; Figure S8. Effects of intramuscularly injected pre-formed fibrillar (PFF) AS on motor phenotype and survival of transgenic M83+/+ PD mice and Figure S9. Mitochondrial respiration and mitochondrial mass in differentiated N2A cells subsequent to eEF2K knockdown. (PDF 2444 kb). [file 40478_2018_554_MOESM1_ESM.pdf]

**Additional File 1 legend**

**Table S1.** Control and PD cases; **Figure S1.** Melanin bleaching in postmortem midbrain sections and immunostaining for phospho-eEF2 (p-eEF2, Thr56); **Figure S2.** Immunostaining for phospho-eEF2 (p-eEF2, Thr56) and phospho-AS (p-ASyn, Ser129) in postmortem control midbrain sections; **Figure S3.** Immunostaining for phospho-eEF2 (p-eEF2, Thr56) and phospho-AS (p-ASyn, Ser129) in postmortem PD midbrain sections; **Figure S4.** Detection of phospho-eEF2 (p-eEF2, Thr56) and phospho-AS (p-ASyn, Ser129) in postmortem control and PD midbrain sections by immunofluorescence; **Figure S5.** Immunostaining for phospho-eEF2 (p-eEF2, Thr56) and phospho-AS (p-ASyn, Ser129) in postmortem control hippocampus sections; **Figure S6.** Immunostaining for phospho-eEF2 (p-eEF2, Thr56) in postmortem PD hippocampus sections- Panoramic views; **Figure S7.** Immunostaining for phospho-eEF2 (p-eEF2, Thr56) and phospho-AS (p-ASyn, Ser129) in postmortem PD hippocampus sections; **Figure S8.** Effects of intramuscularly injected pre-formed fibrillar (PFF) AS on motor phenotype and survival of transgenic M83$^{+/+}$ PD mice and **Figure S9.** Mitochondrial respiration and mitochondrial mass in differentiated N2A cells subsequent to eEF2K knockdown.

**Supplementary Material Jan et al.**

**Contents:**

**1. Table S1**……………………………………… pp1

**2. Supplementary figure legends**……………pp2-3

**3. Supplementary figures**…………………….pp4-12

**Supplementary Figures Legends**

**Figure S1. Melanin bleaching in postmortem midbrain sections and immunostaining for phospho-eEF2 (p-eEF2, Thr56). (a)** Hemotoxylin and eosin (H&E) staining in postmortem midbrain sections from a control case (control-1) before (left) and after (right) a modified melanin destaining/bleaching protocol (see Materials and Methods) (SN= substantia nigra; scale bar, 100µm). **(b)** IHC analysis of phospho-eEF2 (p-eEF2, Thr56) in postmortem midbrain sections from a PD case (PD-2) before (left) and after (right) a modified melanin destaining/bleaching protocol with hemotoxylin as a counterstain. The arrows point to neuromelanin positive cells in substantia nigra (SN), without melanin removal (scale bar, 100µm).

**Figure S2. Immunostaining for phospho-eEF2 (p-eEF2, Thr56) and phospho-AS (p-ASyn, Ser129) in postmortem control midbrain sections. (a-b)** p-eEF2 (T56) and p-ASyn (S129) IHC in postmortem midbrain sections from two control cases (Control- 2 and 3)- see Table S1. (SN- substantia nigra; PAG- periaqueductal gray matter; scale bar, 100µm).

**Figure S3. Immunostaining for phospho-eEF2 (p-eEF2, Thr56) and phospho-AS (p-ASyn, Ser129) in postmortem PD midbrain sections. (a-b)** p-eEF2 (T56) and p-ASyn (S129) IHC in postmortem midbrain sections from four PD cases (PD- 3, 4, 5 and 6)- see Table S1. IHC staining for p-eEF2 is seen in midbrain neurons and glial cells. Lewy body inclusions and neurites (p-ASyn, S129) are seen in all PD cases, except PD-4 in which only a few cells with p-ASyn, S129 IHC staining were detected. (SN- substantia nigra; PAG- periaqueductal gray matter; scale bar, 100µm).

**Figure S4. Detection of phospho-eEF2 (p-eEF2, Thr56) and phospho-AS (p-ASyn, Ser129) in postmortem control and PD midbrain sections by immunofluorescence. (a-b)** p-eEF2 (T56) and p-ASyn (S129) immunofluorescence in postmortem midbrain section one control case (a) and three PD cases (b)- see Table S1. Arrows point to cells where immunopositivity for both p-eEF2 (T56) and p-ASyn (S129) was detected. (SN- substantia nigra; DAPI was used as a nuclear stain; scale bar, 100µm).

**Figure S5. Immunostaining for phospho-eEF2 (p-eEF2, Thr56) and phospho-AS (p-ASyn, Ser129) in postmortem control hippocampus sections. (a-b)** p-eEF2 (T56) and p-ASyn (S129) IHC in postmortem hippocampus sections from control cases (Control-1,2 and 3; Table S1), panoramic, p-eEF2 (T56) (a) and magnified field views (b) (CA1, CA2 and CA3- hippocampal cornu ammonis fields 1-3; DG- dentate gyrus; scale bar, 100µm).

**Figure S6. Immunostaining for phospho-eEF2 (p-eEF2, Thr56) in postmortem PD hippocampus sections- Panoramic views. (a)** p-eEF2 (T56) IHC in postmortem hippocampus sections from PD

cases (PD-1, 2, 3, 4, 5 and 6; Table S1) (CA1, CA2 and CA3- hippocampal cornu ammonis fields 1-3; DG- dentate gyrus). Please see Fig. S7 for the corresponding magnified field views.

**Figure S7. Immunostaining for phospho-eEF2 (p-eEF2, Thr56) and phospho-AS (p-ASyn, Ser129) in postmortem PD hippocampus sections. (a)** p-eEF2 (T56) and p-ASyn (S129) IHC in postmortem hippocampus sections from PD cases (PD-1, 2; Table S1). Only CA3 and GD fields are shown here, and field views for areas CA1-CA2 are presented in the Fig. 2b (CA1, CA2 and CA3- hippocampal cornu ammonis fields 1-3; DG- dentate gyrus; scale bar, 100µm). **(b)** p-eEF2 (T56) and p-ASyn (S129) IHC in postmortem hippocampus sections from PD cases (PD-3, 4, 5 and 6; Table S1). IHC staining for p-eEF2 (T56) is seen predominantly in CA1 and CA2 neurons, and also in CA3 neurons in PD-3. Dentate gyrus in all 6 PD cases did not show any remarkable p-eEF2 (T56) immunopositivity. Lewy body inclusions and neurites (p-ASyn, S129) are seen extensively in CA3 and dentate gyrus in PD-1, while predominantly in CA2 in PD-3, PD-4 and PD-6. (CA1, CA2 and CA3- hippocampal cornu ammonis fields 1-3; DG- dentate gyrus; scale bar, 100µm).

**Figure S8. Effects of intramuscularly injected pre-formed fibrillar (PFF) AS on motor phenotype and survival of transgenic M83$^{+/+}$ PD mice. (a-b)** Foot drop/hindlimb paralysis (a) and hindlimb clasping dystonic phenotype (b) in transgenic M83$^{+/+}$ PD mice intramuscularly (IM) injected bilaterally with phosphate buffered saline (PBS) or pre-formed fibrillar (PFF) mouse wild type AS. **(c)** Hindlimb clasping score in transgenic M83$^{+/+}$ PD mice intramuscularly (IM) injected bilaterally with phosphate buffered saline (PBS) or pre-formed fibrillar (PFF) mouse wild type AS. Clasping score was determined over 10 sec at different time points post-injection until sacrifice (n=10/group; Two-Way ANOVA, *p<0.05, ***p<0.005; error bars indicate Mean ± S.D.). **(d)** Kaplan–Meier survival plot showing post-injection survival time of transgenic M83$^{+/+}$ PD mice intramuscularly (IM) injected bilaterally with phosphate buffered saline (PBS) or pre-formed fibrillar (PFF) mouse wild type AS (n=10/group). Median time to moribund state (foot drop/paralysis) for PFF AS injected mice was 66 days post-injection, while PBS-injected mice remained asymptomatic/disease free over the duration of the experiment.

**Figure S9. Mitochondrial respiration and mitochondrial mass in differentiated N2A cells subsequent to eEF2K knockdown. (a)** Analysis of the Seahorse Mito stress test (oxygen consumption rate-OCR) in control and eEF2K kd cells using oligomycin (2 µM), FCCP (0.5 µM) and rotenone/antimycin A (0.5 µM). The bar graph shows different components of oxygen consumption and respiratory activity (n=18 from three independent experiments; Unpaired T-test, **= p<0.01, NS= not significant; error bars indicate Mean ± S.D.). **(b-c)** Flow cytometry (FACS) analysis of mitochondrial mass by Mitotracker Green dye labeling (b) and mitochondrial DNA-mtDNA (c) in control and eEF2K kd cells (n=12 in e; n=6 in f; two independent experiments; Mann-Whitney test; NS= not significant; error bars indicate Mean ± S.D.).

Fig. S1

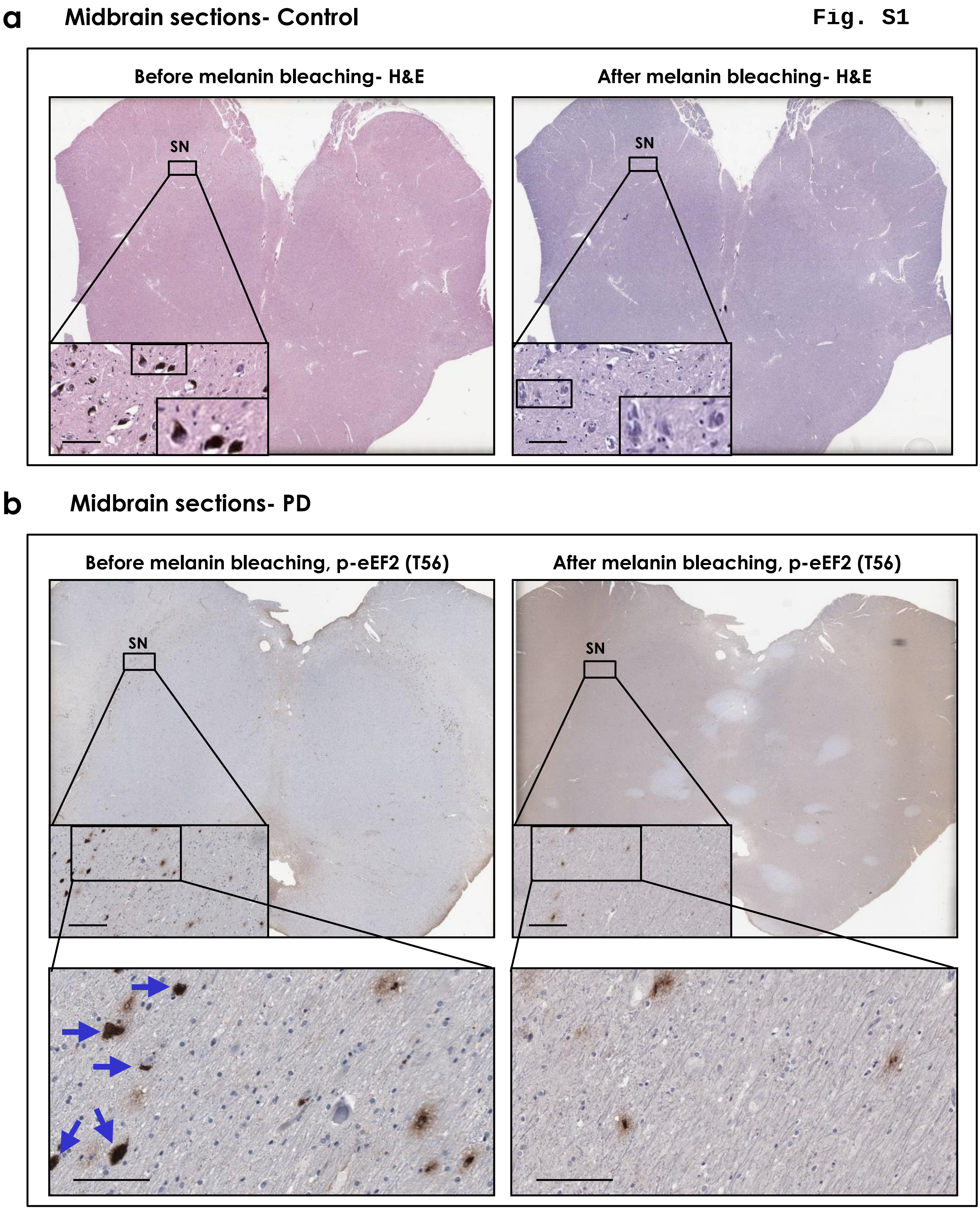

a
Midbrain sections- Control
Before melanin bleaching- H&E
After melanin bleaching- H&E
SN
SN
b
Midbrain sections- PD
Before melanin bleaching, p-eEF2 (T56)
After melanin bleaching, p-eEF2 (T56)
SN
SN

**a** Control midbrain- panoramic and field views

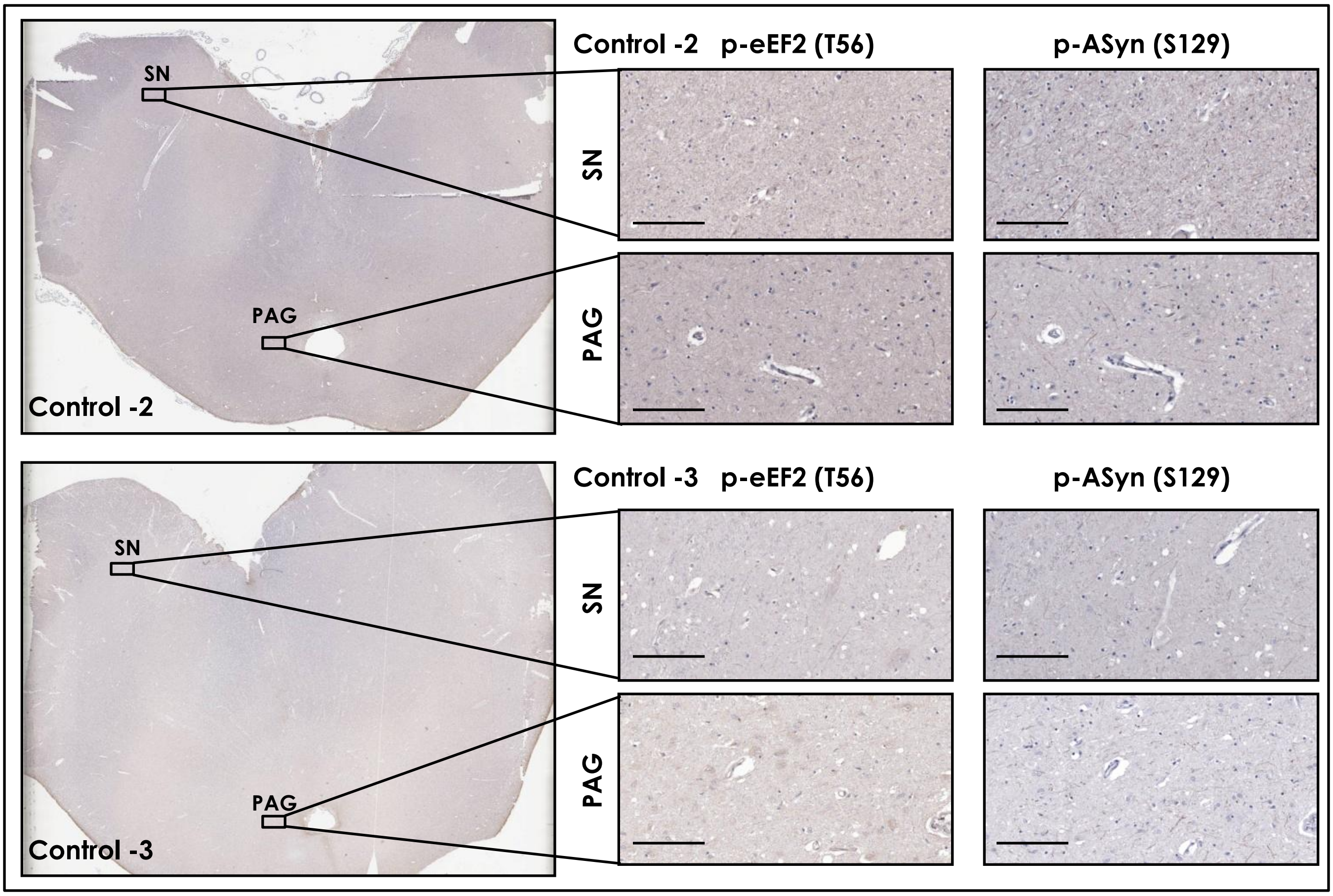

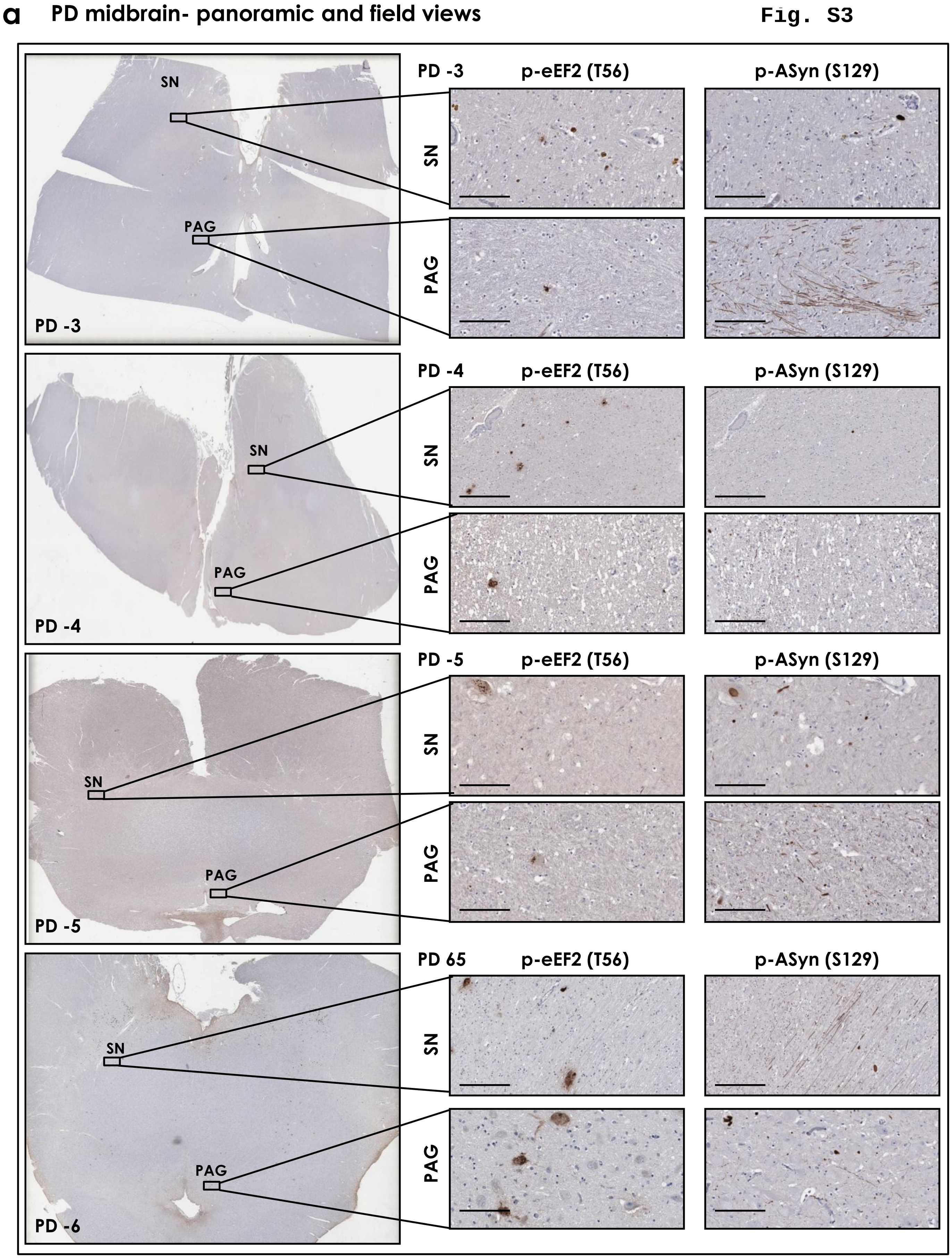
a
PD midbrain- panoramic and field views
PD -3
p-eEF2 (T56)
p-ASyn (S129)
SN
PAG
PD -3
PD -4
p-eEF2 (T56)
p-ASyn (S129)
SN
PAG
PD -4
PD -5
p-eEF2 (T56)
p-ASyn (S129)
SN
PAG
PD -5
PD 65
p-eEF2 (T56)
p-ASyn (S129)
SN
PAG
PD -6

Fig. S4

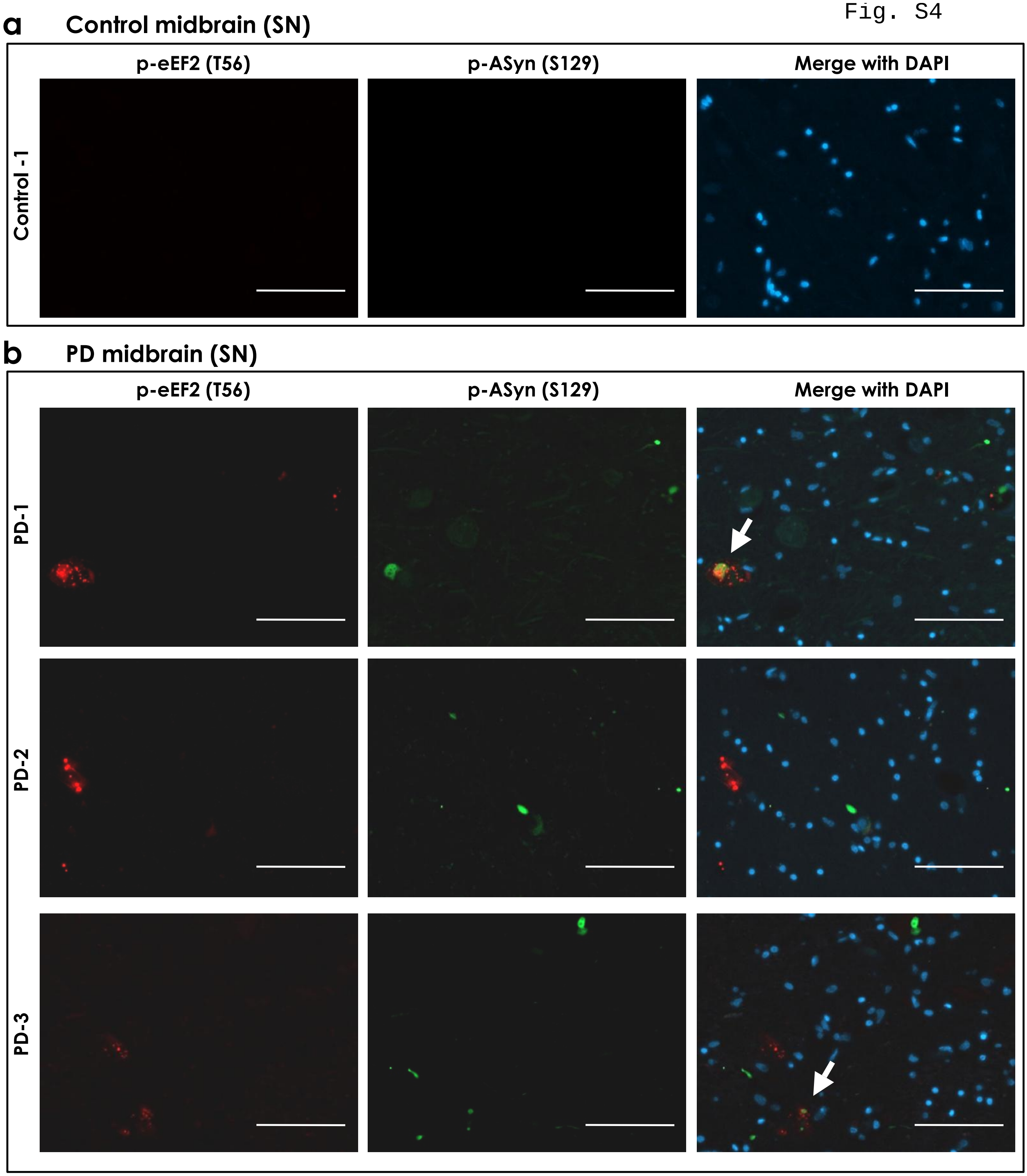

a
Control midbrain (SN)
p-eEF2 (T56)
p-ASyn (S129)
Merge with DAPI
Control -1
b
PD midbrain (SN)
p-eEF2 (T56)
p-ASyn (S129)
Merge with DAPI
PD-1
PD-2
PD-3

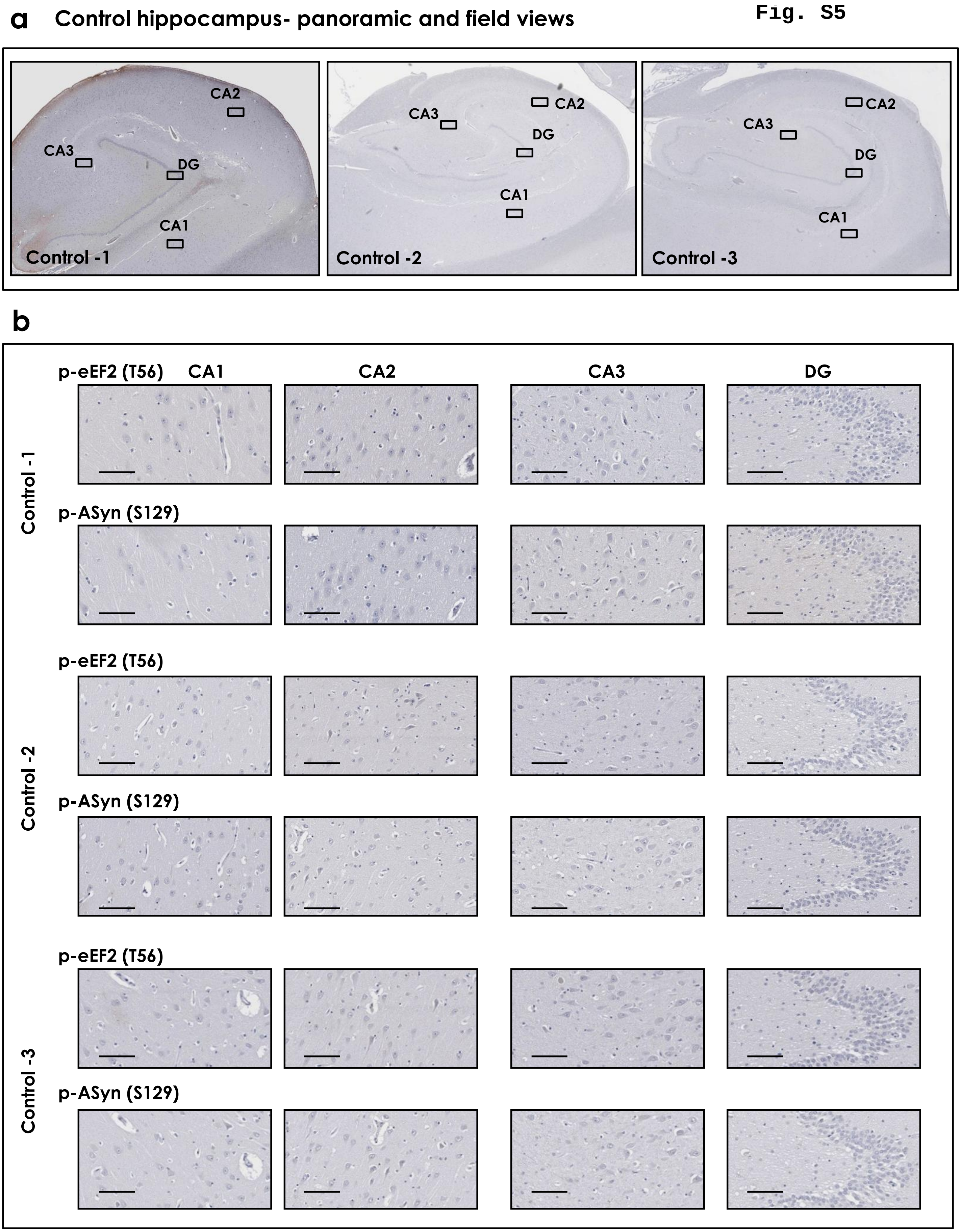
a
Control hippocampus- panoramic and field views
Fig. S5
CA2
CA3
DG
CA1
Control -1
CA3
CA2
DG
CA1
Control -2
CA3
CA2
DG
CA1
Control -3
b
p-eEF2 (T56)
CA1
CA2
CA3
DG
p-ASyn (S129)
Control -1
p-eEF2 (T56)
p-ASyn (S129)
Control -2
p-eEF2 (T56)
p-ASyn (S129)
Control -3

**a** PD hippocampus- panoramic views

Fig. S6

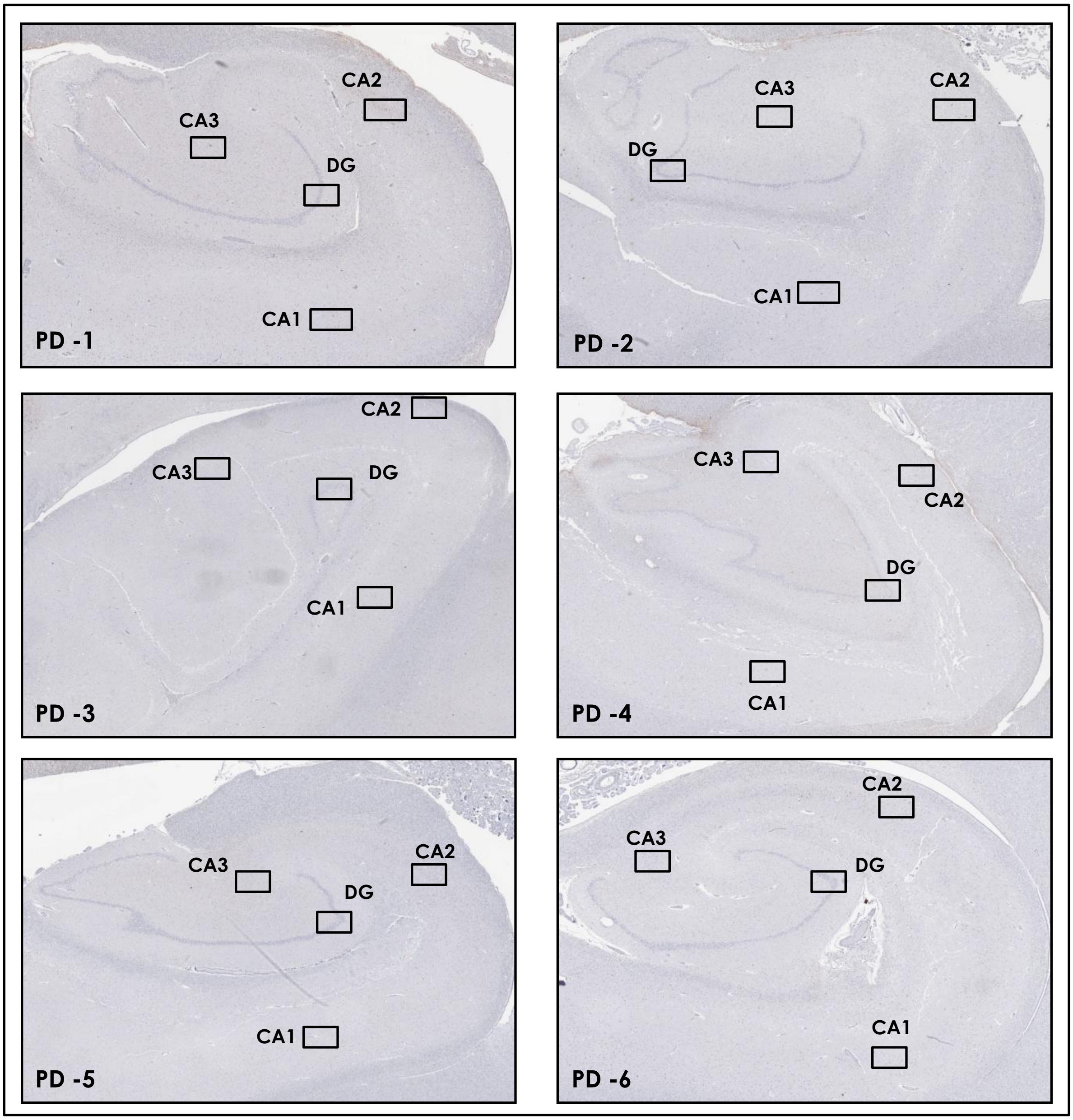

Fig. S7

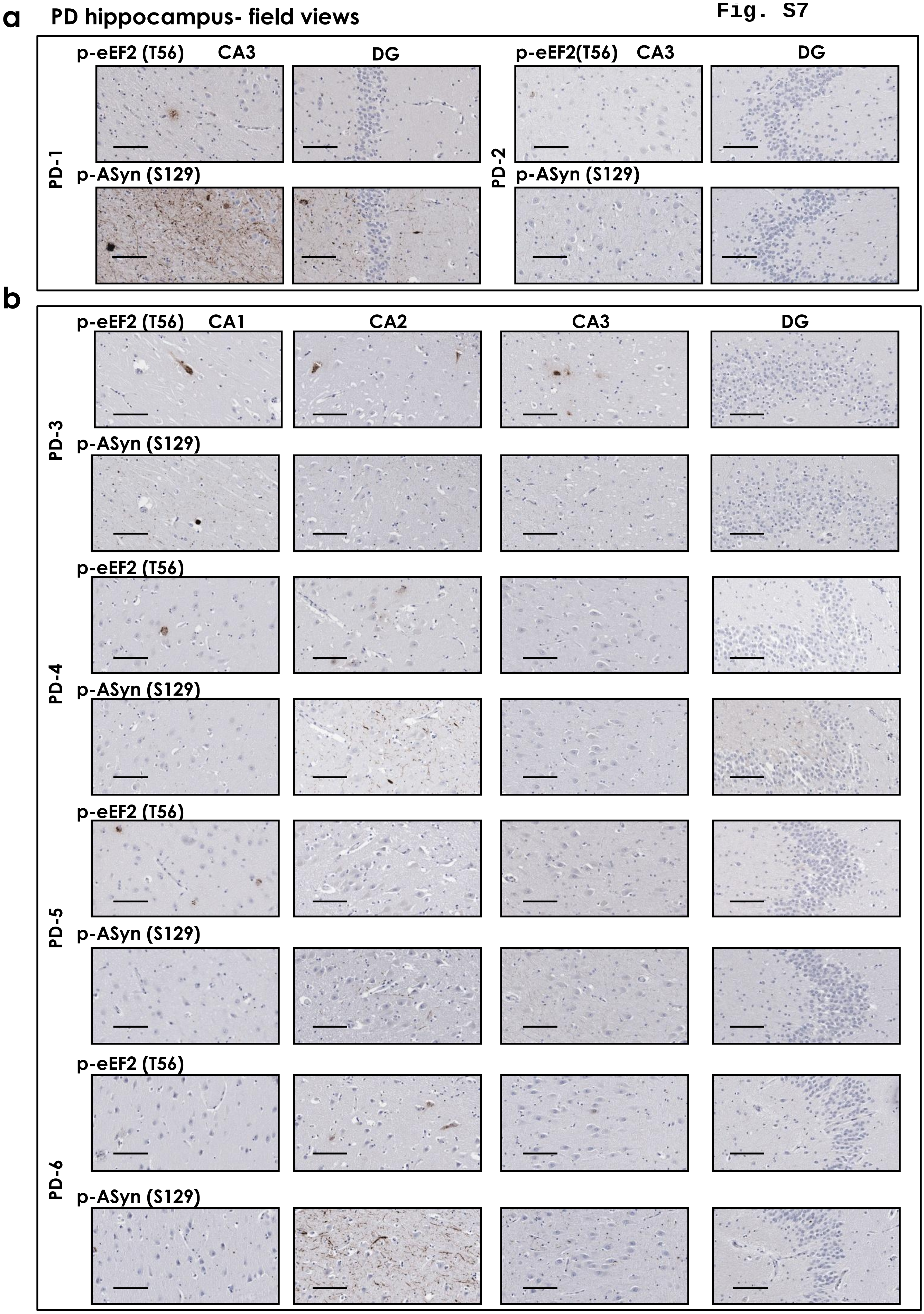

a
PD hippocampus- field views
p-eEF2 (T56)
CA3
DG
PD-1
p-ASyn (S129)
p-eEF2(T56)
CA3
DG
PD-2
p-ASyn (S129)
b
p-eEF2 (T56)
CA1
CA2
CA3
DG
PD-3
p-ASyn (S129)
p-eEF2 (T56)
PD-4
p-ASyn (S129)
p-eEF2 (T56)
PD-5
p-ASyn (S129)
p-eEF2 (T56)
PD-6
p-ASyn (S129)

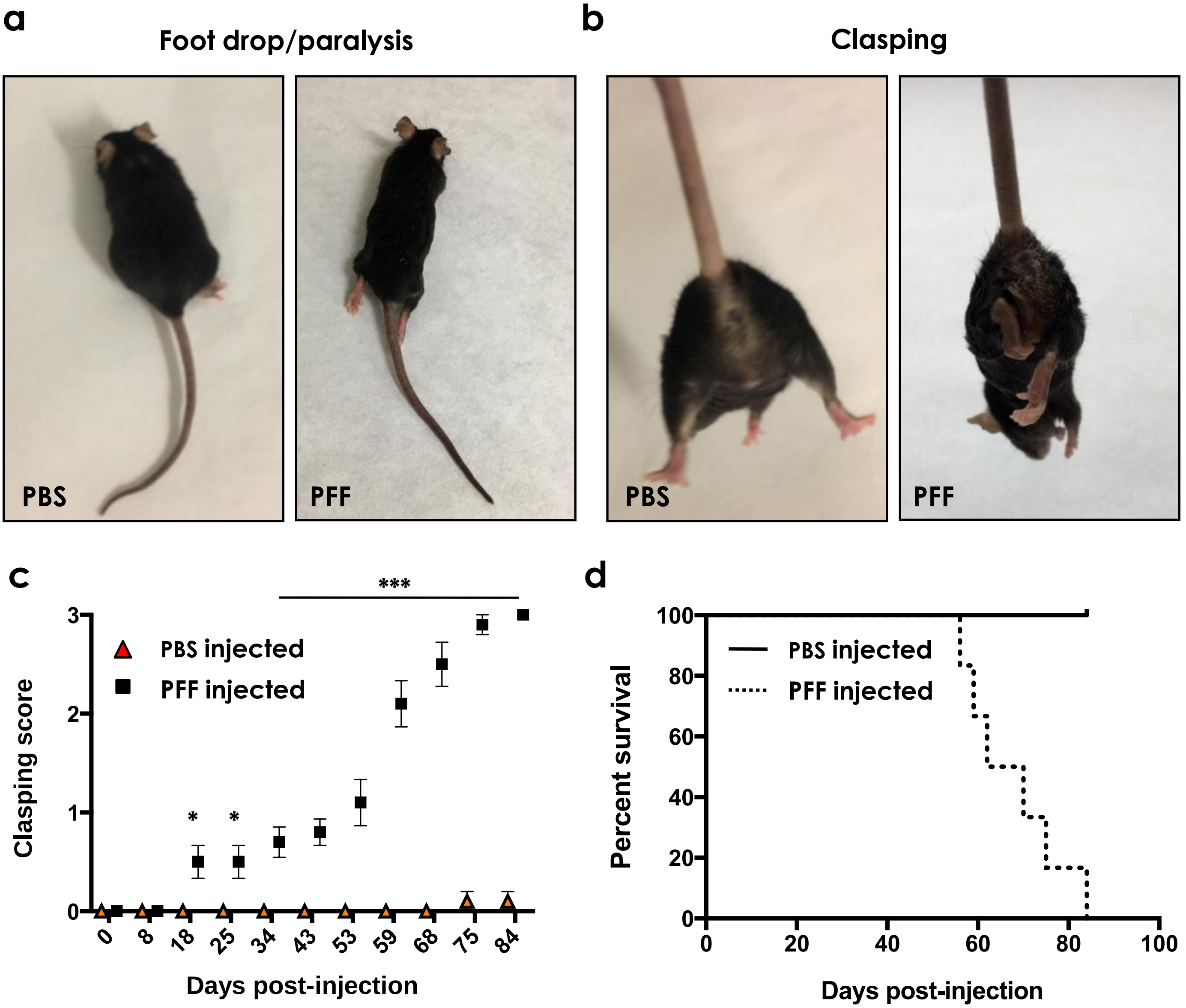
a
Foot drop/paralysis
PBS
PFF
b
Clasping
PBS
PFF
c
***
Clasping score
PBS injected
PFF injected
*
*
Days post-injection
0
8
18
25
34
43
53
59
68
75
84
d
Percent survival
PBS injected
PFF injected
Days post-injection
0
20
40
60
80
100

Fig. S9

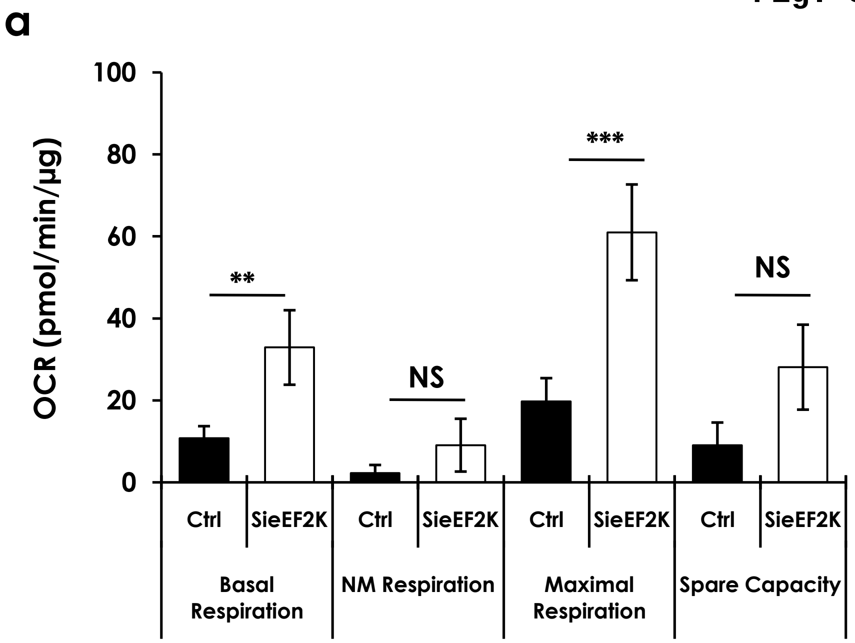
a
100
80
60
40
20
0
OCR (pmol/min/µg)
**
NS
***
NS
Ctrl
SieEF2K
Ctrl
SieEF2K
Ctrl
SieEF2K
Ctrl
SieEF2K
Basal Respiration
NM Respiration
Maximal Respiration
Spare Capacity

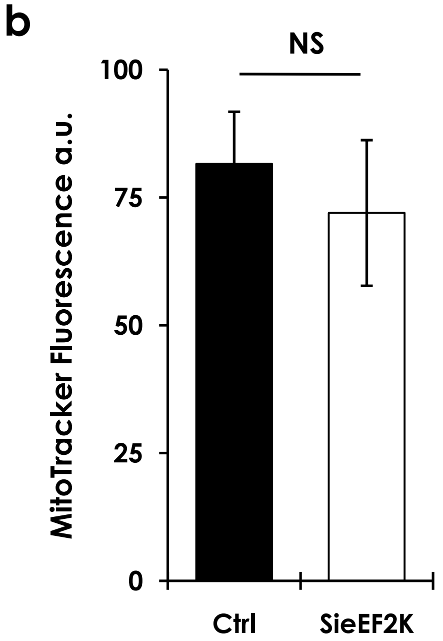
b
NS
100
75
50
25
0
MitoTracker Fluorescence a.u.
Ctrl
SieEF2K

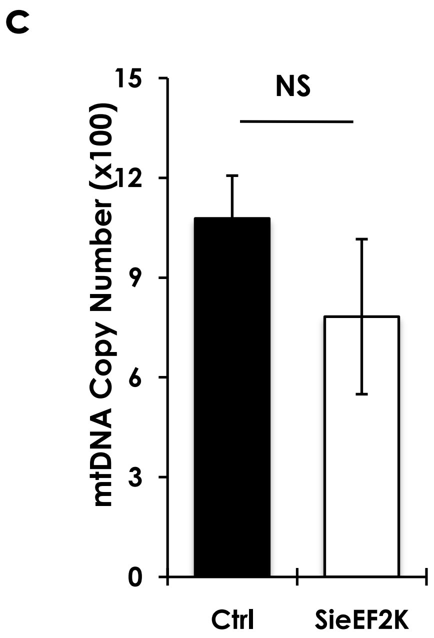
c
NS
15
12
9
6
3
0
mtDNA Copy Number (x100)
Ctrl
SieEF2K
